# Role and therapeutic potential of DEAD-box RNA helicase family in colorectal cancer

**DOI:** 10.3389/fonc.2023.1278282

**Published:** 2023-10-27

**Authors:** Bichun Zheng, Xudong Chen, Qiaoyun Ling, Quan Cheng, Shaoshun Ye

**Affiliations:** Department of Anorectal Surgery, The Affiliated People’s Hospital of Ningbo University, Ningbo, China

**Keywords:** colorectal cancer, DEAD-box RNA helicases, cellular distribution, physiological role, mechanism

## Abstract

Colorectal cancer (CRC) is the third most commonly diagnosed and the second cancer-related death worldwide, leading to more than 0.9 million deaths every year. Unfortunately, this disease is changing rapidly to a younger age, and in a more advanced stage when diagnosed. The DEAD-box RNA helicase proteins are the largest family of RNA helicases so far. They regulate almost every aspect of RNA physiological processes, including RNA transcription, editing, splicing and transport. Aberrant expression and critical roles of the DEAD-box RNA helicase proteins have been found in CRC. In this review, we first summarize the protein structure, cellular distribution, and diverse biological functions of DEAD-box RNA helicases. Then, we discuss the distinct roles of DEAD-box RNA helicase family in CRC and describe the cellular mechanism of actions based on recent studies, with an aim to provide future strategies for the treatment of CRC.

## 1 Introduction

Colorectal cancer (CRC) is the third most commonly diagnosed cancer worldwide, accounting for 10% of all cancers, and is the second most frequent cause of cancer deaths. It is estimated that more than 1.9 million new cases and 0.9 million deaths in 2020 (1). The incidence of CRC is changing rapidly due to its onset at a younger age, and a delayed diagnosis in a more advanced stage (2). Recently, the morbidity increases rapidly in some countries like Spain and countries in east Asia. A transition of dietary habit in these areas may be an important factor. In contrast, the incidence of some high-income countries exhibits downward trend, benefiting from the use of sigmoidoscopy and colonoscopy with polypectomy (3). The risk factors for CRC include smoking, excessive drinking, family history, elder age, male gender, diabetes, obesity, and others. At present, traditional treatments for CRC remain surgery, chemotherapy and radiation therapy. Immunotherapy for CRC has developed fast. Researchers have investigated many immunotherapy methods including monoclonal antibodies, adoptive cell transfer, oncolytic virotherapy, cancer vaccines, cytokines, immune checkpoint inhibitors and other immunotherapeutic agents. Unfortunately, the efficacy is not ideal, especially for patients with advanced stage (3–5). Hence, CRC has become a global public health challenge and it is necessary to seek new therapeutic strategies.

RNA helicases are highly conserved enzymes that play critical roles in RNA metabolism by using adenosine triphosphate (ATP) to bind and modulate RNA structures and ribonucleoprotein (RNP) complexes (6). RNA helicases are divided into multiple subfamilies based on their structural features and the conserved amino acid sequence motifs (6). The DEAD-box RNA helicase family belongs to the helicase superfamily 2 (SF2) and is the largest family of RNA helicases so far. This family is named after its distinctive amino acid sequence [Asp (D)–Glu (E)–Ala (A)–Asp (D)] and contains more than 38 members in humans (7). They regulate almost every aspect of RNA physiological processes, including RNA transcription, editing, splicing and transport (8). In the past several years, studies have found that a large amount of DEAD-box RNA helicases are abnormally expressed and play vital roles in the progression of CRC (**Table 1**). In this review, we first summarize the protein structure, cellular distribution, and diverse biological functions of different DEAD-box RNA helicases. Then, we discuss the distinct roles of DEAD-box RNA helicase family in CRC and describe the cellular mechanism of actions based on recent studies, with an aim to provide future strategies for the treatment of CRC.

**Table 1:** Cellular distributions, intracellular activities and roles in CRC of the DEAD-box proteins.

Symbol	Cellular localization	Intracellular activities	Roles in CRC	Ref
DDX1	Nucleus and cytoplasm	3’-end processing of pre-mRNAs, RNA transport/clearance, regulation of transcription and translation, and modulation of NF-κB transcriptional activity, DNA repair, innate immunity response	promote proliferation, migration, invasion	(7, 9–12)
DDX2A (EIF4A1)	Cytoplasm	regulating cap-dependent translation initiation	promote growth, migration, invasion and metastasis	(13–15)
DDX2B (EIF4A2)	Cytoplasm	miRNA-mediated translational repression	promote tumor growth and lung metastasis, oxaliplatin resistance	(16, 17)
DDX5	Cytoplasm, nucleus	pre-mRNA splicing/alternative splicing, biogenesis of miRNA and ribosomes, regulation of transcription as a transcription factor (coactivators or cosuppressors), NMD, interaction with long noncoding RNAs	promote proliferation, survival, metastasis, migration, invasion, and chemoresistance	(7, 18–21)
DDX17	Cytoplasm, nucleus	pre-mRNA splicing/alternative splicing, biogenesis of miRNA and ribosomes, regulation of transcription as a transcription factor (coactivators or cosuppressors), NMD, interaction with long noncoding RNAs	promote CRC cell proliferation, metastasis and EMT	(7, 18, 22, 23)
DDX6	P-bodies in somatic, stem and tumor cells; nucleus and cytoplasm in oocytes and spermatogenic cells	P-body formation, mRNA storage, mRNA decay, stress granule assembly, translational repression, translational promotion, and miRNA pathways	promote proliferation, cell cycle progression, and Warburg effect, inhibit apoptosis	(24–28)
DDX10	Nucleolar, nucleoplasm and cytoplasmic inclusions, nucleus,	ribosome biogenesis, transcription regulation, alternative splicing, related to MAPK and Akt/NF-κB pathways	promote proliferation, migration, invasion, metastasis, might mediate the immune response in CRC	(29–32)
DDX21	Nucleolus, nucleoplasm, cytoplasm	ribosome biogenesis, RNA splicing, transcription, regulation of innate immunity	promote proliferation, cell cycle progression, invasion, migration, and metastasis inhibit apoptosis, induce genome instability	(8, 33–39)
DDX27	Nucleolus	rRNA maturation, the activation of NF-κB and ERK pathways	promote proliferation, migration, invasion, EMT, stemness and chemoresistance, inhibit apoptosis	(40–45)
DDX39B	Nucleus, cytoplasm	mRNA splicing and nuclear export, translation, promotion of virus replication, telomere protection	promote proliferation, migration, invasion, cell cycle progression, metastasis, and Warburg effect	(46–51)
DDX46	Nucleus	pre-mRNA splicing, promotion of virus replication	promote proliferation, inhibit apoptosis	(52–54)
EIF4A3	Nucleus and cytoplasm	mRNA splicing, RNA trafficking and nonsense-mediated mRNA decay, translation initiation	promote proliferation, invasion, migration, angiogenesis, stemness and oxaliplatin resistance as an RBP	(55–61)
DDX54	Nuclear speckles, cytoplasm	transcription co-activator and co-repressor, pre-mRNA splicing, regulating mRNA stability	promote proliferation, migration, invasion and EMT	(62–67)
DDX56	Nucleolus, cytoplasm	ribosome biogenesis, ribosome assembly, transcription, pre-mRNA splicing, miRNA-mediated post-transcriptional regulation	promote proliferation and cell cycle progression	(68–73)
DDX60	Cytoplasm	antiviral, intestinal immune response, cancer immunotherapy and radiosensitivity	positively regulate MHC-I expression	(74–77)
DDX3X	Cytoplasm, nucleus, organelles (centrosome, mitochondria), intracellular RNA/protein bodies	pre-mRNA splicing, transcription, mRNA export, translation, innate immunity response, embryo development, cellular stress response	confusing	(78–82)
DDX58	Cytoplasm	antiviral, inflammation, cancer development, autophagy, apoptosis, and classic Singleton–Merten syndrome	confusing	(83–86)

## 2 Structural features of DEAD-box RNA helicase family proteins

All DEAD-box helicase family members contain a profoundly conserved helicase core that is flanked by domains of variable sequences of amino (N)- and carboxy (C)-terminal ends. The core element possesses the binding sites for ATP and RNA. The variable auxiliary domains are responsible for the various functions of these proteins, because they interact with RNA or protein components of substrates.

### 2.1 The conserved helicase core

**Figure 1:**
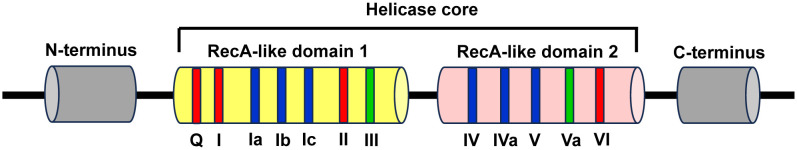
Schematic representation of the structural features of DEAD-box RNA helicase family proteins. The DEAD-box RNA helicase family proteins contain a conserved helicase core and variable N- and C-terminus regions (grey). The conserved helicase core consists of two RecA-like domains (domain 1 and domain 2), which contain 7 and 5 conserved sequence motifs, respectively. Among these motifs, the motifs Q, I, II, and VI are involved in ATP binding and hydrolysis (red), the motifs of Ia, Ib, Ic, IV, IVa, and V are participated in RNA recognition (blue), and the motifs of III and Va are involved in the coordination between RNA and ATP binding (green).

The conserved helicase core is composed of two recombinase A (RecA)-like domains named domain 1 and domain 2, which form the ATP- and RNA-binding clefts and contribute to ATP binding and hydrolysis, RNA binding and duplex unwinding. These two domains contain at least 12 profoundly conserved sequence motifs, with motifs Q, I, Ia, Ib, Ic, II/DEAD, and III in the N-terminal domain (domain 1, D1) and IV, IVa, V, Va, and VI in the C-terminal domain (domain 2, D2). Among them, the motifs Q, I, II, and VI are involved in ATP binding and hydrolysis, the motifs of Ia, Ib, Ic, IV, IVa, and V are participated in RNA recognition, and the motifs of III and Va are involved in the communication between RNA and ATP binding (**Figure 1**) (7, 87).

The two RecA-like domains are connected by flexible central hinge region that allows changing their orientation to each other. Therefore, DEAD-box helicases can switch their conformations between “inactive open”, wherein both domains move freely as for each other in the absence of ATP and RNA, and “active closed”, wherein a compact helicase core structure is formed when collaboratively binds with ATP and RNA. The alteration of conformations plays a vital role for the functions of DEAD-box helicases (7, 88).

DEAD-box RNA helicase family proteins are specific for ATP to catalyze RNA duplex unwinding and RNA-induced hydrolysis of the triphosphate. This effect is mediated by the particular interaction between the base-pairing face of the adenine nucleotide and a conserved glutamine of the Q motif. However, RNA binds over the two helicase core domains opposite the ATP-binding site. The RNA-binding motif consists of a highly and positively charged surface cleft that can bind single-stranded RNA (ssRNA) with five or more nucleotides. The RNA-binding sites only contact the sugar phosphate backbone of the RNA substrates, which makes the DEAD-box helicases function as general RNA chaperones targeting various RNAs (7, 88).

### 2.2 The variable auxiliary domains

The helicase core of the DEAD-box helicases is flanked by variable N- and C- terminal regions. The length varies from a few to several hundred amino acids. The auxiliary domains can interact with particular RNAs or protein cofactors, which contribute to the function variety in this protein family (89). The length and composition of the variable auxiliary domains have been provided by Michael et al. (90). However, the specific roles of these auxiliary domains in most DEAD-box RNA helicases are still largely unknown.

## 3 Cellular localization and physiological role of DEAD-box RNA helicase family proteins

DEAD-box RNA helicases are multifunctional proteins and involved in a wide range of cellular processes, including transcription, translation, pre-mRNA splicing, RNA degradation, microRNA (miRNA) biogenesis, gene regulation, rRNA processing and ribosome biogenesis (91). The cellular localization of DEAD-box RNA helicases is closely related to their functions.

### 3.1 DDX1

The DDX1 refers to the Dead-Box 1 protein which is predominantly located to the nucleus of normal cells, while relocation of DDX1 from the nucleus to the cytoplasm is observed in specific situations (92, 93). Importantly, the presence of DDX1 in the cytoplasm is related to tumorigenesis (93). DDX1 has been demonstrated to participate in multiple cellular processes, such as DNA repair, 3’-end processing of pre-mRNAs, RNA transport/clearance, regulation of transcription and translation, and modulation of NF-κB transcriptional activity (7). Moreover, DDX1 is associated with DNA repair, in which DDX1 promotes the removal of RNA-DNA structures and homologous recombination at DNA double-strand breaks (DSBs) (9). Additionally, DDX1 forms complex with DDX21 and DDX36 and binds to the adaptor protein TRIF to sense dsRNA, leading to the activation of innate immune signaling [reviewed in (10)].

### 3.2 EIF4A proteins

In mammals, three EIF4A proteins have been characterized: EIF4A1 (also known as DDX2A), EIF4A2 (also known as DDX2B) and EIF4A3 (also known as DDX48). The sequences of these proteins share high homology. EIF4A1 and EIF4A2 are cytoplasmic-localized and there is approximately 90% sequence identity at the amino acid level between them (94). EIF4A3 is a nucleocytoplasmic shuttling protein that is found both in nucleus and cytoplasm. It shares 60% similarity with EIF4A1. In some extent, the three EIF4A proteins can unwind dsRNA *in vitro*. However, they are inefficient helicases and their helicase activities are largely dependent on the binding partners (94). For example, when EIF4A interacts with eIF4G and eIF4B, the RNA helicase and ATPase activities of EIF4A increases more than 100-fold (95). However, eIF4H is not able to stimulate the RNA helicase activity of EIF4A (95).

EIF4A1 is thought to function primarily as an important component of eIF4F complex, which also contains the mRNA cap-binding protein eIF4E and the scaffolding protein eIF4G. EIF4A1 functions in regulating the initiation of cap-dependent translation that is the most vital step of protein production (13). The 40S ribosomal subunit is recruited by the interactions among eIF4F complex, eIF3, and the 40S subunit before the binding of eIF4E to the 5’ cap of the mRNA. Then, this 43S pre-initiation complex scans the 5’-untranslated region (UTR) for the AUG initiation codon. During scanning, EIF4A1 is able to unwind the stable secondary structures of mRNA at the 5’-UTR in an ATP-dependent manner. EIF4A1 is necessary for the translation initiation rates of a variety of pro-oncogenic mRNAs, leading to tumorigenesis and progression (96, 97). EIF4A1 has been demonstrated to be aberrantly regulated in many types of cancers, such as CRC, gastric cancer, breast cancer, cervical cancer, and hepatocellular carcinoma (13).

Although the protein sequences of EIF4A1 and EIF4A2 are highly similar, they are functionally distinct. EIF4A1 is essential for translation initiation, while EIF4A2 is not necessary for this process. The binding partners of both proteins are divergent. EIF4A1 predominantly binds to eIF4G, whereas EIF4A2 preferentially binds to cNOT7, a member of CCR4-NOT complex, which plays an important role in miRNA-mediated translational repression (16, 94).

EIF4A3 is a core component of the exon junction complex (EJC) (i.e., the Y14/Magoh heterodimer and MLN51) (13). Physiologically, EIF4A3 is involved in post-transcriptional gene regulation by facilitating EJC control of pre-mRNA splicing and monitor mRNA quality before translation to influence nonsense-mediated mRNA decay (NMD), mRNA localization, and translation (13, 55, 56). EIF4A3 can be recruited by long non-coding RNAs (lncRNAs) to decrease its aggregation around RNAs, which influences the translation of target genes (55). In addition, EIF4A3 serves as a specific translation initiation factor for nuclear cap-binding complex-dependent translation (57).

### 3.3 DDX3

DDX3 contains two genes DDX3X and DDX3Y, which locate on the X- and Y-chromosome, respectively (98). Because DDX3X escapes X-chromosome inactivation, females carry two active alleles and males only have one. DDX3Y is the Y chromosome-linked homolog only carried by males. The sequences of their nucleic acid and amino acid share 88% and 91% homology in human. The differences between them are largely distributed in their N-terminal domains. Both proteins possess nuclear export sequence (NES) and nuclear localization sequence (NLS), which makes DDX3 can shuttle from the nucleus to the cytoplasm by binding to CRM1 and TAP (98, 99). If the leucine residues in this region mutate, DDX3 will accumulate in the nucleus. In the nucleus, DDX3X can be recruited to E-cadherin promoter, leading to the repression of its expression and tumorigenesis (100). DDX3X can also localize to the centrosome and mitochondria, intracellular RNA/protein bodies (such as stress granules) (98). DDX3X functions largely dependent on their binding partners and intracellular localization (98). A recent comprehensive review has summarized the biological functions of DDX3X. These functions include pre-mRNA splicing, transcription, mRNA export, translation, innate immunity response, and embryo development (78). In addition, DDX3X participates in cellular stress response, in which DDX3X is recruited by stress granules that suppress the NLRP3 inflammasome activation (101).

Although DDX3Y protein is ubiquitously expressed in mouse, it is widely expressed in many tissue types but not translated, except in testis in human. Human DDX3Y is also found in leukemia and lymphoma cells, but not in normal B lymphocytes (98, 102). Studies have demonstrated that DDX3X and DDX3Y are functionally redundant (98).

### 3.4 DDX5 (also known as p68) and DDX17 (also known as p72)

DDX5 (p68) and DDX17 (p72) share the greatest homology among the members of DEAD-box RNA helicase family, sharing approximately 90% homology across the conserved helicase core. However, the N- and C-terminal regions of both proteins are highly divergent (about 60% and 30% sequence similarity respectively). DDX5 and DDX17 can interact with each other to form heterodimers. It appears that they have several partially redundant functions although they also have distinct roles (103). Both proteins are nuclear and cytoplasmic shuttle proteins and expressed in most tissues and cells (18). DDX5 and DDX17 performs nucleocytoplasmic shuttling by the classic Ran GTPase-dependent pathway and an exportin/importin-dependent pathway, respectively. They help other proteins carry out nucleocytoplasmic shuttling (18).

They exhibit RNA-dependent ATP hydrolase activity and RNA helicase activity as well as RNA annealing activity. Both proteins are involved in multiple cellular processes, including pre-mRNA splicing/alternative splicing, biogenesis of miRNA and ribosomes, regulation of transcription as a transcription factor (coactivators or cosuppressors), NMD and interaction with long noncoding RNAs [reviewed in (7, 18)]. DDX5 and DDX17 are subject to multiple post-translational modifications, which play vital roles in regulating protein interactions and functions. They can undergo acetylation, ubiquitination, and sumoylaton. Moreover, DDX5 can also be modified by phosphorylation, methylation, and O-GlcNAcylation. Different post-translational modifications endow them with diverse biological functions, and the same modification at different sites causes disparate functional outcomes. Diverse post-translational modifications have been found in many tumors and they are involved in tumorigenesis and tumor progression (18). In most instances, DDX5 phosphorylation facilitates cell proliferation and invasion; the acetylation of DDX5/DDX17 enhances their transcriptional coactivation abilities; K48 ubiquitination is responsible for DDX5 degradation; K63-polyubiquitination of DDX17 regulates miRNA biogenesis and histone modification, leading to the increase of cancer stem-like features; sumoylation elevates the stability of DDX5/DDX17 and promotes their inhibitory transcriptional activity. O-GlcNAcylation of DDX5 facilitates tumor development; and methylation of DDX5 is related to R-loop resolution (18, 104).

### 3.5 DDX6

DDX6 is located to the processing bodies (P-bodies) in somatic, stem and tumor cells (24). It is essential for the assembly and maintenance of P-bodies, which are membrane-less cytoplasmic organelles generated by phase-separation when mRNAs and neighboring RNA binding proteins (RBPs) assemble into ribonuclear particle (RNP) as granules and are thought to be the place to store and decay the translationally inactive mRNAs (105, 106). A variety of translational repression and decapping-related proteins, miRNA-induced silencing complex (RISC) components, and translationally repressed mRNAs are accumulated in P-bodies. P-bodies are associated with the control of post-transcriptional processes. Stefano et al. (105) found that once DDX6 lost its activity, P-bodies would disassemble and release their contents to impact cell fate. DDX6 exhibits multiple functions in regulating RNA metabolism, including P-body formation, mRNA storage, mRNA decay, stress granule assembly, translational repression, translational promotion, and miRNA pathways (106). The divergent functions of DDX6 may be involved in its associated complexes (107). In addition, DDX6 localizes to both the nucleus and cytoplasm in oocytes and spermatogenic cells and may participate in diverse functions in these cells (25, 26).

### 3.6 DDX10

Nucleolar DDX10 is involved in early stages of ribosome biogenesis as a component of the small subunit processome and it is essential for 18S ribosomal RNA (rRNA) production (29, 30). However, α-synuclein (α-Syn) interacts with DDX10 and sequesters it to the nucleoplasm and cytoplasmic inclusions to increase cytotoxicity via facilitating α-Syn oligomerization and decreasing α-Syn fibril formation (30). DDX10 can fuse with NUP98 to form NUP98-DDX10. NUP98-DDX10 protein mainly localizes to distinct punctate structures within the nucleus. This fusion protein deregulates gene expression, in which the helicase motif VI of DDX10 plays a crucial role, at the transcriptional level (31). Zhou and coworkers have found that DDX10 can also regulate alternative mRNA splicing of RPL35 (32). Downregulation of DDX10 inhibits MAPK signaling pathway to reduce the proliferation, invasion and migration of osteosarcoma cells (108), but facilitates ovarian cancer cell proliferation through Akt/NF-κB pathway (109).

### 3.7 DDX21 and DDX50

DDX21 is predominantly localized to nucleolus. In the nucleolus, DDX21 is associated with ribosome biogenesis, which is largely through regulation of rDNA transcription by interacting with small nucleolar RNAs (snoRNAs) to regulate rRNA processing and modification. This process is dependent on its ADP-ribosylation (33). Interestingly, DDX21 re-localizes from the nucleolus to the nucleoplasm under specific conditions, such as the treatment with PARP inhibitor or glucose elevation (33, 34). In the nucleoplasm, DDX21 interacts with RNA splicing factors to assemble into larger protein complexes to regulate the splicing of some genes (34). After virus infection, DDX21 is cleaved by Caspase-3/6 at D126 and then translocates from the nucleus to the cytoplasm. Cytoplasmic DDX21 negatively regulates the IFN-β signaling pathway via inhibiting the formation of the DDX1-DDX21-DHX36 complex (35, 36).

DDX50 (also known as RHII/Guβ) is a paralogue of DDX21, sharing 55.6% amino acid identity (110). Similarly, DDX50 shows a predominant nucleolar localization, while it can translocate from the nucleolus to nucleoplasm by cytotoxic drug treatment (111). DDX5 and DDX21 have non-redundant roles. It is likely that in the nucleoplasm, DDX50 is a co-factor for c-Jun-activated transcription (111). DDX50 can inhibit the replication and dissemination of virus by activating the transcription of IFN genes (110, 112). However, the exact role of nucleolar DDX50 remains unknown.

### 3.8 DDX27

DDX27 is a nucleolar protein and it regulates the maturation of rRNA, which is required for ribosome biogenesis and the translation of genes (40, 41). DDX27 is also related to the activation of NF-κB and ERK pathways (42).

### 3.9 DDX39A and DDX39B

DDX39A (also known as URH49 and DDX39) and DDX39B (also known as UAP56) show a high degree of homology, with 90% in amino acid identity in humans (46). Both proteins can shuttle between the nucleus and the cytoplasm (46), and exhibit similar or redundant functions in mammalian cells. They participate in various cellular processes, including mRNA splicing, nuclear export, and translation (47, 48). Moreover, DDX39A and DDX39B are required for some virus replication and are involved in nuclear export of virus mRNAs and prevention of dsRNA accumulation in infected cells (49). What’s more, sumoylation of DDX39A alters the binding and export of antiviral transcripts to facilitate virus escape from innate immunity (113). Additionally, DDX39A is also required for telomere protection to maintain global genome integrity (47).

### 3.10 DDX42 and DDX46

DDX42 (also known as SF3b125) and DDX46 (also known as PRPF5) are enriched in nucleus especially Cajal bodies and associated with human U2 snRNP, which participates in early spliceosome assembly (114). The 17S U2 is the functional form of human U2 snRNP and its core components contain the SF3b complex, SF3a complex, 12S U2 core (U2 snRNA, Sm ring, U2-A’ and U2-B”), the splicing factor TAT-SF1 and DDX46 (115). DDX42, which has RNA chaperone activity, binds to the SF3b complex, but it is released after accomplishing its role in U2 snRNP assembly. DDX46 is a component of 17S U2 snRNP and it plays an important role in pre-mRNA splicing, functions during assembly of pre-spliceosome and proofreading of the branch site (52, 114). Moreover, DDX42 and DDX46 play different roles in antiviral innate immunity. Endogenous DDX42 inhibits HIV-1 replication independently of the interferon response (116). Whereas, nuclear DDX46 promotes VSV replication by inhibiting the production of type I interferons (53).

### 3.11 DDX54

DDX54 is distributed in nuclear speckles (62). It interacts with estrogen receptors and suppresses their transcriptional activity (63). DDX54 is a constitutive androstane receptor (CAR)-binding protein and it is a gene (or promoter)-selective co-activator for CAR (62). During DNA damage response, DDX54 is required for the splicing efficacy of its target pre-mRNAs (64). Additionally, DDX54 also functions in cytoplasm. It can regulate mRNA stability by directly binding or acting as an RBP of lncRNAs (65, 66).

### 3.12 DDX56

DDX56 is a nucleolar protein and participates in ribosome biogenesis and ribosome assembly (68, 69). Furthermore, DDX56 is involved in transcription (70), pre-mRNA splicing (71), as well as miRNA-mediated post-transcriptional regulation by degradation of primary miRNA (72). Additionally, DDX56 is related to antiviral innate immunity. It performs different roles under different virus infections, which are associated with its cellular localization. During some virus (eg chikungunya virus, pseudorabies virus) infection, DDX56 re-localizes from the nucleus to the cytoplasm. In the cytoplasm, DDX56 acts as an antiviral factor by binding and destabilizing the incoming viral genomic RNA to control the earliest step of viral replication or by regulating the IFN-β signaling pathway to inhibit virus replication (68, 73). It seems that in the nucleus, DDX56 directly interacts with viral proteins or indirectly with host proteins to promote the replication of some viruses (e.g. alphavirus, West Nile virus) (117).

### 3.13 DDX58/RIG-I

DDX58 encodes RIG-I, which is mainly localized to cytoplasm. DDX58 is firstly identified as a pattern recognition receptor in host innate immunity that recognizes cytosolic viral nucleic acids to activate the IFN signaling and proinflammatory cytokines, resulting in the promotion of host antiviral immune response (83). DDX58 can also act as an RBP to activate NF-κB signaling by stabilizing p65 mRNA (83, 118). Recent studies have demonstrated that DDX58 is involved in multiple physiological processes, including viral infection, inflammation, cancer development, autophagy, apoptosis, and classic Singleton–Merten syndrome (83, 84).

### 3.14 DDX60

DDX60 is an IFN-inducible cytoplasmic helicase and plays an important role in human antiviral activity. As an upstream factor of RIG-I, DDX60 not only activates RIG-I signaling in a ligand-specific manner but also is involved in RIG-I-independent viral RNA degradation. This indicates that DDX60 is a sentinel for cytoplasmic antiviral response (74). Additionally, DDX60 selectively reduces translation of viral type II internal ribosome entry sites (75). Recent studies have shown that DDX60 is involved in intestinal immune response and modulates cancer immunotherapy and radiosensitivity (76).

## 4 Roles of DEAD-box proteins in colorectal cancer

The majority of DEAD-box proteins play tumor-promoting roles in CRC. DDX60 seems to have the tumor-suppressing role in CRC. However, we observed that DDX3X and DDX58 exert both tumor-promoting and tumor-suppressing roles in CRC.

### 4.1 Members with tumor-promoting effects

#### 4.1.1 DDX1

DDX1 protein was highly expressed in human CRC specimens. However, its level was not correlated with patients’ pathological grade and clinical stage (11). Consistently, DDX1 mRNA expression was elevated in CRC cell lines. Using CRISPR/Cas9-mediated genome editing to disrupt DDX1 gene led to decrease of cell proliferation, suppression of sphere-forming capacity and expression of cancer stem cell marker genes (LGR5, CD133, ALDH1 and SOX2) in CRC cells as well as smaller solid tumor size and lower proliferative marker PCNA expression in xenograft tumor in nude mice (11). DDX1 functioned via directly promoting the transcription of LGR5 by interacting with the -1837 to -1662 region of the enhancer/promoter region of LGR5 (11). Likewise, overexpression of DDX1 promoted, while its knockdown decreased migration and invasion ability of CRC cells (12). What’s more, DDX1 was essential for circLONP2-enhanced migration and invasion ability of CRC cells through directly interaction (12).

#### 4.1.2 DDX2

DDX2 contains two subtypes, including DDX2A (EIF4A1) and DDX2B (EIF4A2). Li et al. (14) found that EIF4A1 mRNA was significantly increased in primary CRC tissues compared to the adjacent normal tissues, and EIF4A1 protein was overexpressed in 86% (44/51) of tumor samples. Overexpression of EIF4A1 could abort miR-133a-suppressed CRC cell growth (14). Söylemez and his coworkers (119) showed that compared with non-cancer subjects, EIF4A1 mRNA expression was decreased in the tumor tissues from CRC patients of stage I, III, and IV, but increased in stage II. However, the mRNA level of EIF4A1 was increased in peripheral blood of patients in stages I, II, and III, but reduced in stage IV (119). Long noncoding RNA MK5-AS1, which promoted the migration, invasion and metastasis of CRC, functioned partially through recruitment RBM4 and EIF4A1 to facilitate MK5 translation. MK5 further phosphorylated c-Jun, leading to the activation of SNAI1 transcription by directly binding to its promoter (15).

Both mRNA and protein levels of EIF4A2 was upregulated in CRC tumors. High EIF4A2 predicted patients’ poor survival. Knock-down or small-molecule inhibitor of EIF4A2 inhibited the invasion, migration and stemness, and elevated oxaliplatin sensitivity in CRC cells (17). What’s more, genetic knockdown of EIF4A2 suppressed tumor growth and lung metastasis in cell-derived xenograft mouse model. Silvestrol, an inhibitor of EIF4A2, improved oxaliplatin sensitivity in patient-derived xenograft mouse model (17). Mechanically, EIF4A2 as a transcriptional target of ZNF143 might function via mediating the translation of c-Myc, an oncoprotein that is deregulated in more than 50% human cancers and is related to cancer cell aggressive phenotypes, and drug resistance (17, 120).

#### 4.1.3 DDX5 (p68) and DDX17 (p72)

**Figure 2:**
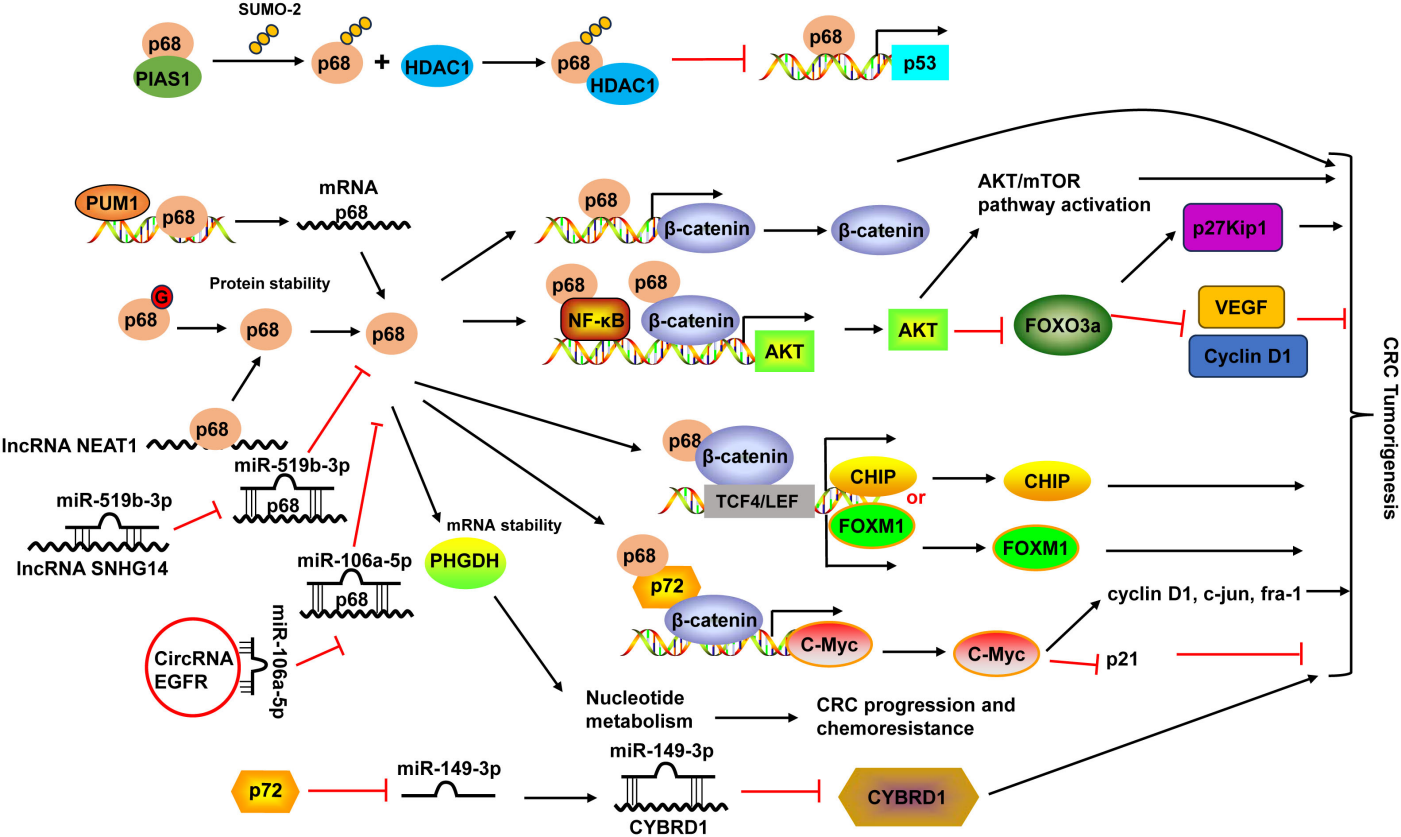
Schematic diagram of the DDX5 (also known as p68) and DDX17 (also known as p72) in CRC development. PIAS1 interacted with p68 and enhanced p68 sumoylation by SUMO-2. Sumoylated p68 increased the transcriptional repression activity of p68 and suppressed its ability to function as a coactivator of p53 by interacting with HDAC1. p68 protein could be modified by O-GlcNAcylation (G) to maintain its protein stability. p68 not only acted as a transcriptional co-activator of β-catenin, it also occupied AKT promoter with β-catenin as well as NF-κB and cooperated with them to potentiate AKT transcription, leading to the increases of AKT mRNA and protein levels, and consequent activation of Akt/mTOR pathway and inhibition of FOXO3a pathway. Moreover, p68 along with β-catenin occupied TCF4/LEF binding sites on the promoter of endogenous CHIP and FOXM1 and modulated their transcription. p68 could also mediate the mRNA stabilization of PHGDH, leading to nucleotide metabolic reprogramming to promote CRC progression and chemoresistance. p68 expression can be regulated by PUM1 and many non-coding RNAs, including lncRNA NEAT1 and SNHG14, and circular RNA EGFR. p68 and p72 formed complexes with β-catenin and augmented β-catenin to activate the transcription of proto-oncogenes, including c-Myc, cyclin D1, c-jun, and fra-1, and suppress the transcription of the cell cycle inhibitor p21(WAF1/CIP1), which is mediated via c-Myc. p72 downregulated miR-149-3p expression, leading to the upregulation of its target CYBRD1 expression to promote the metastasis and EMT of CRC cells. HDAC1, histone deacetylase 1; NF-κB, nuclear factor-κB; PUM1, Pumilio RNA-binding family member 1; VEGF, vascular endothelial growth factor; TCF4, transcription factor 4.

DDX5 (p68) protein was overexpressed and post-translationally modified (predominantly by sumoylation modification) mainly by the small ubiquitin-like modifier-2 (SUMO-2) on a single site (K53) in CRC (121, 122). During this process, PIAS1 acted as an E3 ligase interacting with DDX5 and enhanced its sumoylation by SUMO-2. SUMO modification of DDX5 increased the transcriptional repression activity of DDX5 and suppressed its ability to function as a coactivator of p53, which was mediated by the interaction between sumoylated DDX5 and histone deacetylase 1 (HDAC1) (122). Additionally, DDX5 protein can be modified by O-GlcNAcylation to maintain its protein stability in CRC, leading to the activation of the AKT/mTOR pathway (**Figure 2**) (123).

The high expression of DDX5 was associated with poor prognosis (124). Interestingly, the combination of DDX5 and fructose-bisphosphate aldolase A (ALDOA), which played a key role in glycolysis and gluconeogenesis, had more significant prognostic values in CRC patients (124). DDX5 enhanced cancer cell proliferation and survival as well as metastasis in CRC (19). DDX5 not only acted as a transcriptional co-activator of β-catenin (125), it also occupied AKT promoter with β-catenin as well as nuclear factor-κB (NF-κB) and cooperated with them to potentiate AKT transcription, leading to the increases of AKT mRNA and protein levels and consequent higher nuclear exclusion and degradation of FOXO3a. The decrease of tumor suppressor FOXO3a led to changes of its target genes, like downregulation of p27Kip1, upregulation of vascular endothelial growth factor (VEGF) and Cyclin D1 (19). Moreover, DDX5 along with β-catenin occupied transcription factor 4 (TCF4)/LEF binding sites on the promoter of endogenous CHIP (STUB1) and FOXM1 and modulated their transcription, leading to enhanced cell proliferation and migration in CRC (20, 126). A recent study demonstrated that DDX5 could mediate the mRNA stabilization of PHGDH, a key enzyme in the serine synthesis pathway, leading to nucleotide metabolic reprogramming to promote CRC progression and chemoresistance (21) (**Figure 2**).

Pumilio RNA-binding family member 1 (PUM1) interacted with DDX5 in 3’-UTR and positively regulated its mRNA expression in CRC cells (127). DDX5 expression could also be regulated by non-coding RNAs. For example, lncRNA nuclear-enriched abundant transcript 1 (NEAT1) directly bound to the DDX5 protein, enhanced its stability in CRC (125). LncRNA SNHG14 promoted CRC cell proliferation and invasion as a sponge for miR-519b-3p to regulate its target gene DDX5 (128). Circular RNA EGFR enhanced DDX5 expression via sponging miR-106a-5p to fulfill its oncogenic functions in CRC (129) (**Figure 2**).

DDX17 and DDX5 share the greatest homology among the DDX family members and they can form heterodimers to modulate various physiological and pathological processes (18). Similar to DDX5, DDX17 levels were upregulated in CRC tissues (22, 23). Higher DDX17 protein expression was positively related to larger tumor size, lymph node invasion, distant organ metastasis and advanced AJCC stage, as well as liver metastasis (23). Patients with higher DDX17 expression exhibited shorter overall survival, and it was an independent prognostic factor for patients’ overall survival (23). In CRC, DDX5 and DDX17 formed complexes with β-catenin and augmented β-catenin to activate the transcription of proto-oncogenes, including c-Myc, cyclin D1, c-jun, and fra-1, and suppress the transcription of the cell cycle inhibitor p21(WAF1/CIP1), which is mediated via c-Myc, resulted in the promotion of CRC cell proliferation and tumor formation (22). DDX17 can also function independent of DDX5 in CRC. DDX17 contributed to the metastasis and epithelial to mesenchymal transition (EMT) of CRC cells by downregulating miR-149-3p expression, leading to the upregulation of its target CYBRD1 expression (23) (**Figure 2**).

#### 4.1.4 DDX6

**Figure 3:**
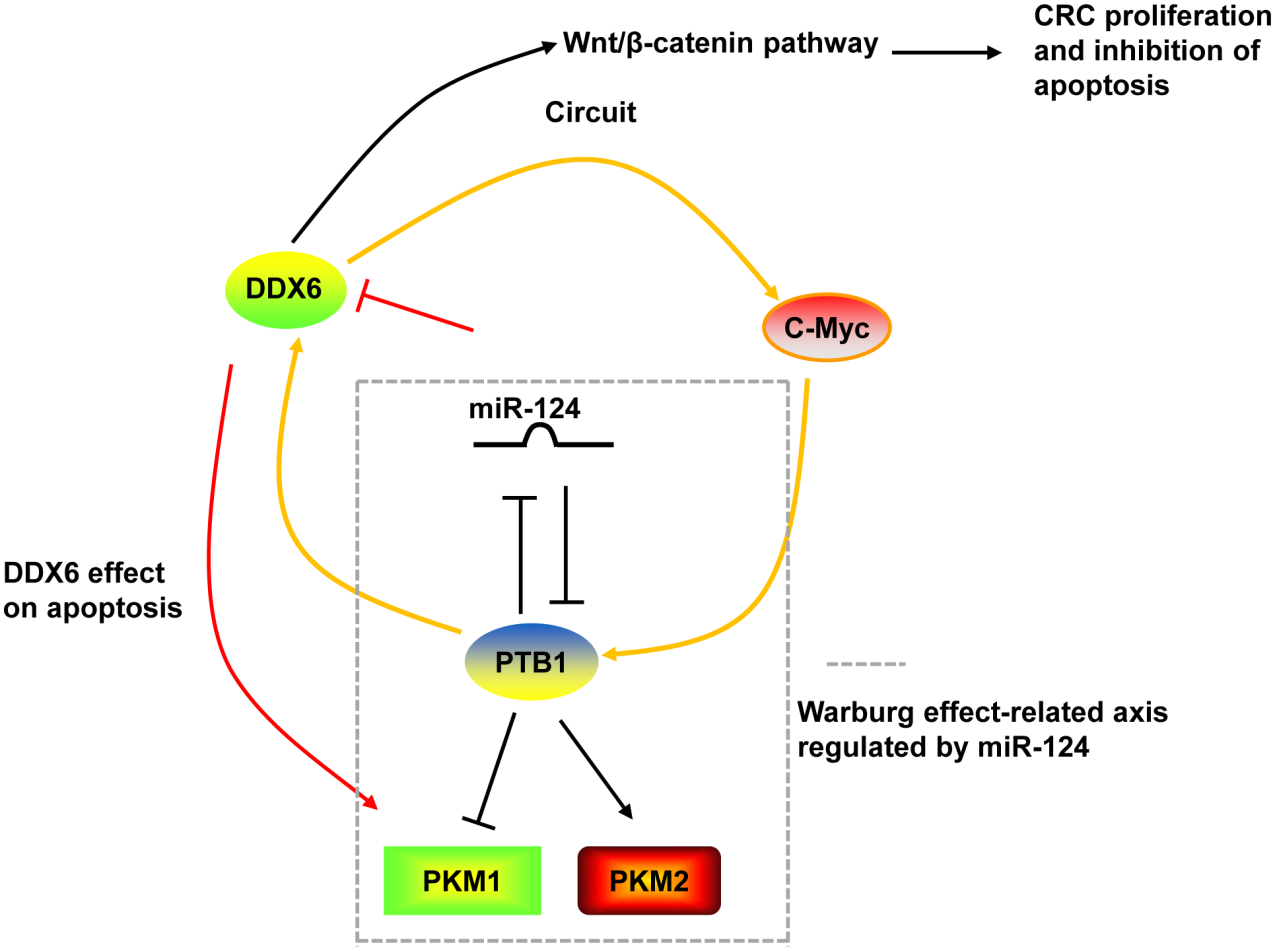
The mechanism of DDX6 in CRC. DDX6 could activate the canonical Wnt/β-catenin pathway to promote the proliferation and inhibit the apoptosis of CRC cells. DDX6 also affect the apoptosis of CRC cells via upregulating PKM1 expression. Additionally, DDX6 facilitated c-Myc translation and contributed to the Warburg effect through the DDX6/c-Myc/PTB1 positive-feedback circuit. DDX6 was a target of miR-124. And miR-124 also targeted PTB1 and regulated the Warburg effect by switching the expression of PKM isoform from PKM2 to PKM1.

DDX6 was overexpressed in tumor tissues of CRC (130, 131). Downregulation of DDX6 reduced the viability, caused cell cycle arrest in S phase, induced apoptosis, and decreased the Warburg effect in CRC cells and suppressed tumor growth in nude mice (27, 28). The growth inhibition and induction of apoptosis by DDX6 knockdown were through the downregulation of PKM1 and the suppression of canonical Wnt/β-catenin pathway (27, 28). On the other hand, DDX6 possessed RNA unwinding activity toward c-Myc RNA and facilitated c-Myc translation via unfolding the structure of internal ribosomal entry site (IRES) of c-Myc (28, 131). DDX6 contributed to the Warburg effect through the DDX6/c-Myc/PTB1 positive-feedback circuit. DDX6 was a target of miR-124. miR-124 also targeted PTB1, which is a splicer of the PKM gene, and regulated the Warburg effect by switching the expression of PKM isoform from PKM2 to PKM1 (28) (**Figure 3**).

#### 4.1.5 DDX10

The mRNA and protein expression levels of DDX10 were both highly expressed in tumor tissues of CRC. High expression of DDX10 was associated with the poor prognosis of patients. Downregulation of DDX10 not only suppressed cell growth and colony formation, inhibited migration and invasion of CRC cells *in vitro*, but also prevented tumor growth and lung metastasis in nude mice (32). GO/KEGG enrichment analysis revealed that DDX10 was positively related to microtubule binding, the cell cycle and RNA splicing. Zhou et al. (32) demonstrated that DDX10 might modulate the occurrence and metastasis of CRC via regulating the E2F pathway after alternatively splicing RPL35 mRNA. Unfortunately, they did not clearly clarify the mechanism of DDX10 by wet-experiments but only analyzed by bioinformatics (32). Interestingly, there was a strong correlation between DDX10 expression and the number of infiltrating immune cells, including CD4^+^ T cells, CD8^+^ T cells and macrophages, in colon adenocarcinoma (COAD), which was analyzed by the TIMER database (32). This indicated that DDX10 might mediate the immune response in CRC. The exact mechanism of DDX10 in CRC needs further study.

#### 4.1.6 DDX21

DDX21 was highly expressed in human CRC (37–39, 132–134). Its expression was associated with CRC cancer stages (133). Upregulation of DDX21 was related to shorter overall survival and disease-free survival in stage III and IV CRC patients (8), but was correlated with the longer survival in early stage of CRC patients with microsatellite instability (134). Xie et al. (37) revealed that DDX21 possessed an oncogenic activity, as indicated by the decreases of colony formation and cell proliferation abilities in CRC cells with DDX21 knockdown. Aberrantly high expression of DDX21 caused genome fragility by impairing double-strand repair and delaying homologous recombination repair. Most importantly, DDX21-induced genome instability led to high sensitivity to some chemotherapeutic drugs, such as irinotecan, oxaliplatin, etoposide and hydroxyurea, providing a new precision treatment strategy for CRC with high DDX21 expression (37).

**Figure 4:**
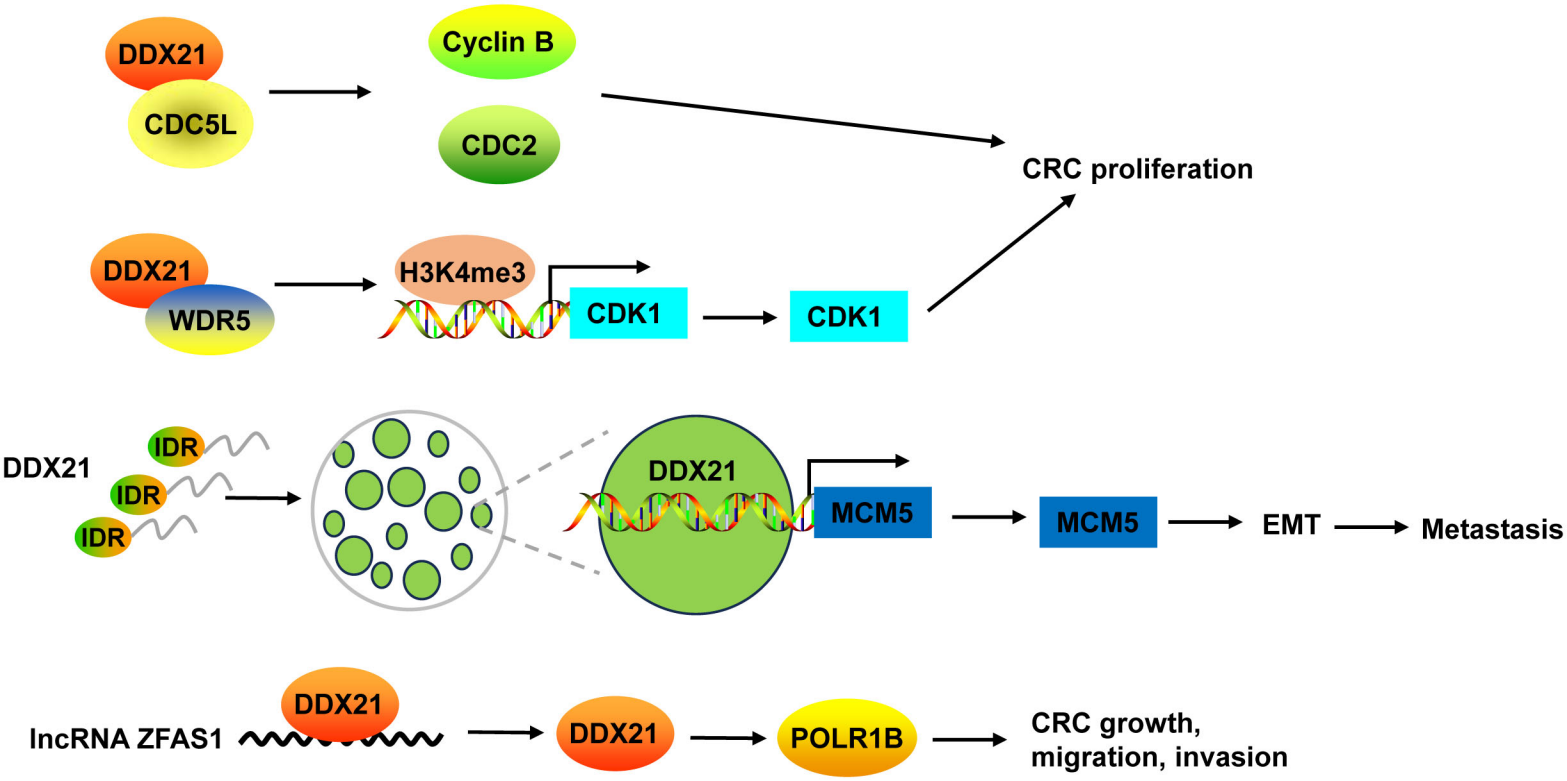
The mechanism of DDX21 in CRC. DDX21 promoted CRC proliferation not only via interacting with CDC5L to regulate cyclin B and CDC2, but also by directly recruiting WDR5 to activate CDK1 gene expression through enhancing H3K4me3 on its promoter. DDX21 has a strong IDR, which makes it form phase-separated condensates. Phase separation of DDX21 facilitated CRC metastasis via directly targeting on MCM5 to induce its expression and subsequently leading to the activation of EMT pathway. LncRNA ZFAS1 directly recruited DDX21 protein and positively regulated its expression, and then affected POLR1B expression, resulting in promotion of CRC cell growth, migration and invasion. CDC5L, cell division cycle 5-like; H3K4me3, trimethylation of histone H3 on Lys 4; IDR, intrinsically disordered region.

Inhibition of DDX21 not only inhibited CRC cell proliferation and tumor growth, but also arrested cell cycle at G2/M phase (38, 39). On one hand, DDX21 functioned via interacting with cell division cycle 5-like (CDC5L) to regulate cyclin B and CDC2, G2/M phase marker (38). On the other hand, DDX21 directly recruited WDR5, the core component of the MLL/SET1 methyltransferase complex, to enhance trimethylation of histone H3 on Lys 4 (H3K4me3) on the CDK1 promoter to activate CDK1 gene expression (39) (**Figure 4**).

Recently, Gao and co-workers found that DDX21 promoted CRC cell migration and invasion *in vitro*, and facilitated CRC to liver metastasis and lung metastasis *in vivo* (8). DDX21 has a strong intrinsically disordered region (IDR) at its N-terminal that includes 182 amino acids, which makes it form phase-separated condensates with liquid-like behavior in CRC cells. Phase separation of DDX21 facilitated CRC metastasis via directly targeting on MCM5 to induce its expression and subsequently leading to the activation of EMT pathway (8) (**Figure 4**).

DDX21 protein can be regulated by lncRNA in CRC. For example, lncRNA ZFAS1 directly recruited DDX21 protein through the specific motif (AAGA or CAGA) and positively regulated its expression, and then affected their downstream target gene POLR1B, the critical components of Pol I RNA polymerase complex, resulting in promotion of CRC cell proliferation, invasion, migration, and suppression of cell apoptosis *in vitro* and *in vivo* (135) (**Figure 4**).

#### 4.1.7 DDX27

**Figure 5:**
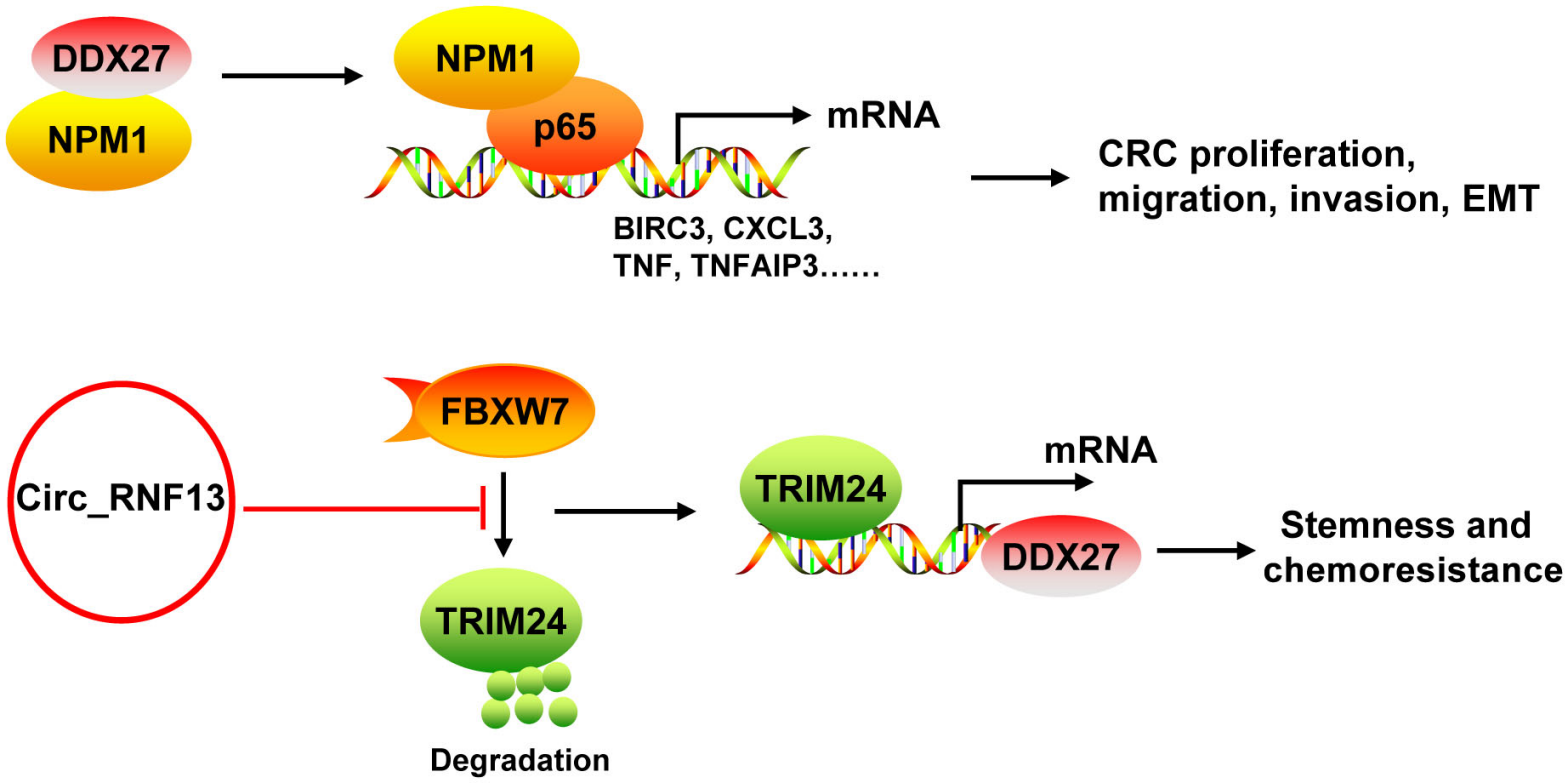
The mechanism of DDX27 in CRC. DDX27 promoted the proliferation, migration, invasion and EMT of CRC cells via directly interacting with NPM1 in the nucleus. Their binding promoted the interaction between nuclear NPM1 and NF-κB p65 to increase the binding activity of p65 to promoters of NF-кB target genes (such as BIRC3, CCL20, CXCL3, NFKBIA, TNF, and TNFAIP3), leading to increases of their transcription. DDX27 could promote CRC stemness and chemoresistance. During these processes, DDX27 expression was positively regulated by circ_RNF13 via TRIM24-mediated transcriptional regulation. And circ_RNF13 stabilized TRIM24 via suppressing FBXW7-mediated TRIM24 degradation. NPM1, nucleophosmin.

DDX27 mRNA and protein levels were upregulated in CRC, which was mainly resulted from the DNA copy number gain (43, 44). DDX27 overexpression was an adverse prognostic factor for CRC patients. DDX27 promoted the proliferation, migration, invasion and EMT, but inhibited apoptosis *in vitro*. Silencing of DDX27 suppressed tumor growth and lung metastasis in subcutaneous CRC xenograft mouse models (43). Mechanistically, DDX27 directly interacted with nucleophosmin (NPM1) in the nucleus, without any influence on the expression and nuclear translocation of NPM1. Their binding promoted the interaction between nuclear NPM1 and NF-κB p65 to increase the binding activity of p65 to promoters of NF-кB target genes (such as BIRC3, CCL20, CXCL3, NFKBIA, TNF, and TNFAIP3), leading to increases of their transcription (43) (**Figure 5**). Moreover, downregulation of DDX27 could weaken the self-renewal ability of cancer stem cells and elevated the differentiation potential as well as promoted chemosensitivity in CRC both *in vitro* and *in vivo* (44, 45). During these processes, DDX27 expression was positively regulated by circ_RNF13 via TRIM24-mediated transcriptional regulation. Circ_RNF13 stabilized TRIM24 via suppressing FBXW7-mediated TRIM24 degradation (45) (**Figure 5**). However, the mechanism DDX27 in stemness and chemoresistance of CRC needs to be further explored (44).

#### 4.1.8 DDX39B

DDX39B expression was abnormally upregulated in CRC tissues and cells (50). Elevated DDX39B protein expression was correlated with aggressive progression and poor prognosis of CRC patients (51). *In vitro* experiments demonstrated that DDX39B enhanced the proliferation, migration and invasion capacities as well as EMT of CRC cells (50, 51). Also, DDX39B drove the cell cycle from G0/G1 phase to the S phase. Consistently, DDX39B deficiency not only inhibited tumor growth in subcutaneous tumor models in nude mice (51), but also suppressed spleen metastases in an orthotopic transplantation model of CRC and lung metastasis in a CRC model via tail-vein injection (50, 51).

**Figure 6:**
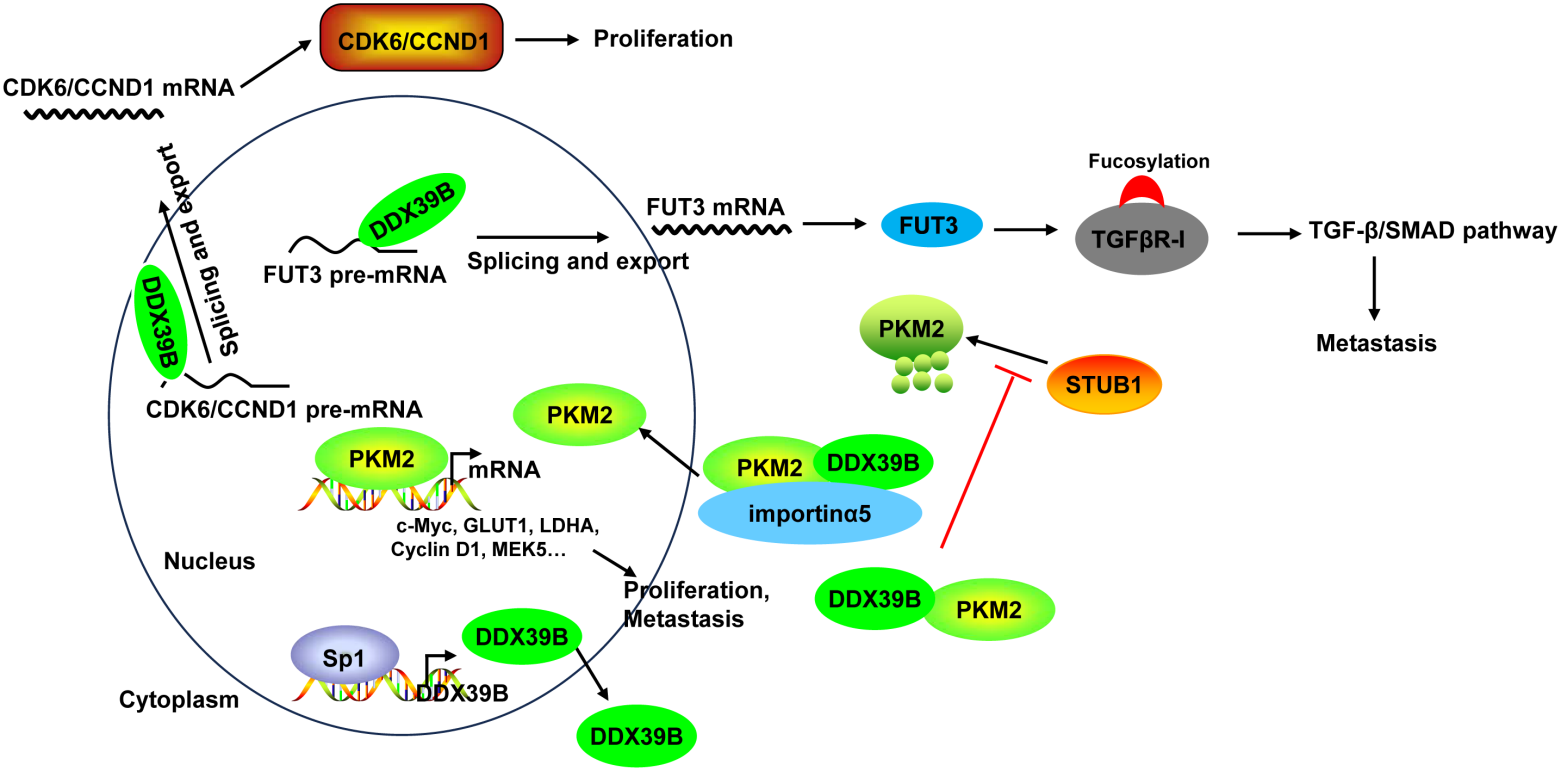
The mechanism of DDX39B in CRC. DDX39B upregulated the expression of FUT3 by regulating mRNA splicing and export, followed by the fucosylation of TGFβR-I and activation of the TGF-β/SMAD signaling pathway to facilitate the metastasis of CRC. Moreover, DDX39B bound directly to CDK6/CCND1 pre-mRNA and subsequently promoted the pre-mRNA splicing and export, leading to the increases of their expression levels, which contributed to CRC cell proliferation. Additionally, DDX39B directly bound to PKM2 to increased its stability by competitively suppressing STUB1-mediated PKM2 ubiquitination and degradation, and then accelerated the nuclear translocation of PKM2 by recruiting importin α5 in an ERK1/2-mediated phosphorylation-independent manner. Nuclear PKM2 promoted the expression of oncogenes and glycolytic genes (such as c-Myc, GLUT1, LDHA, Cyclin D1, and MEK5) to induce CRC cell proliferation and metastasis. DDX39B expression could be induced by Sp1 transcription factor through direct binding.

Mechanistically, DDX39B upregulated the expression of FUT3, a type of fucosyltransferases, by regulating mRNA splicing and export, followed by the fucosylation of TGFβR-I, which subsequently enhanced activation of the TGF-β/SMAD signaling pathway to facilitate the invasion and metastasis of CRC (50). Moreover, DDX39B directly bound to PKM2 relying on the arginine residue 319 of DDX39B and increased the stability of PKM2 by competitively suppressing STUB1-mediated PKM2 ubiquitination and degradation, and then accelerated the nuclear translocation of PKM2 by recruiting importin α5 in an ERK1/2-mediated phosphorylation-independent manner. Nuclear PKM2 functions as a protein kinase and transcriptional coactivator to regulate the expression of oncogenes and glycolytic genes that trigger the Warburg effect (such as c-Myc, GLUT1, LDHA, Cyclin D1, and MEK5), leading to CRC cell proliferation and metastasis (51). Additionally, Zhang et al. (136) found that DDX39B bound directly to the first exon of the CDK6/CCND1 pre-mRNA and subsequently promoted CDK6/CCND1 pre-mRNA splicing and export, leading to the increases of their expression levels, which contributed to CRC cell proliferation. Sp1 transcription factor induced DDX39B transcription by directly binding to the GC box of the DDX39B promoter in CRC cells (51) (**Figure 6**).

#### 4.1.9 DDX46

Although DDX46 protein expression was observed both in human CRC tissues and adjacent tissues, its level was significantly increased in CRC tissues compared with adjacent tissues (54). Downregulation of DDX46 using RNAi lentivirus significantly not only suppressed cell proliferation but also induced apoptosis via increasing the expression of cleaved caspase-3 and PARP in CRC cells. However, the mechanism of DDX46 in CRC remains unclear (54).

#### 4.1.10 EIF4A3

**Figure 7:**
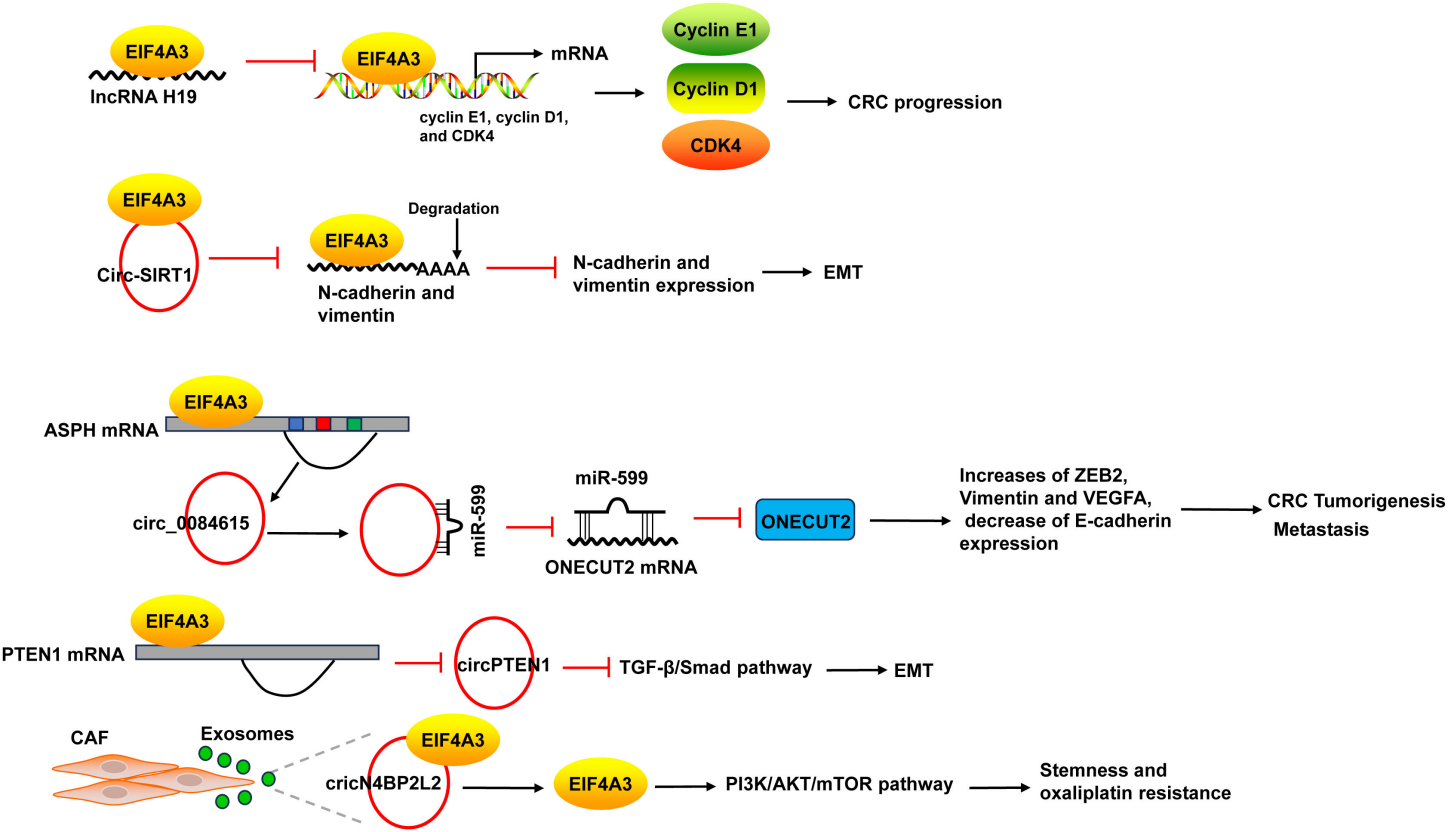
The mechanism of EIF4A3 in CRC. The binding of EIF4A3 and lncRNA H19 obstructed the recruitment of EIF4A3 to the mRNA of cyclin E1, cyclin D1, and CDK4, resulted in their upregulation. The binding between EIF4A3 and circ-SIRT1 decreased the abundance of EIF4A3 at the mRNAs of N-cadherin and Vimentin, leading to the suppression of these mRNAs degradation. EIF4A3 could bind to the flanking sequences of circ_0084615, a circRNA derived from ASPH, and positively regulated circ_0084615 expression. Circ_0084615 functioned in CRC via miR-599/ONECUT2 pathway. While EIF4A3 could inhibit the cyclization of circPTEN1 after binding to its flanking sequences. CircPTEN1 suppressed CRC metastasis via inhibition of TGF-β/Smad-mediated EMT. CAF secreted exosomes cricN4BP2L2 could bind to EIF4A3 and positively regulate its expression, and subsequently caused the activation of PI3K/AKT/mTOR axis in CRC cells, which promoted CRC cells stemness and oxaliplatin resistance. ASPH, aspartate beta-hydroxylase; CAF, cancer-associated fibroblasts.

EIF4A3 acts as an RBP to promote the progression of CRC. The binding of EIF4A3 and lncRNA H19 obstructed the recruitment of EIF4A3 to the mRNA of cyclin E1, cyclin D1, and CDK4, resulted in the upregulation of these genes (137). EIF4A3 could bind to circ-SIRT1, and circ-SIRT1 overexpression did not affect EIF4A3 expression, but decreased the abundance of EIF4A3 at the mRNAs of the EMT marker proteins N-cadherin and Vimentin, thereby blocking the inhibitory effect of EIF4A3 on EMT and promoting the proliferation and invasion of CRC cells (58) (**Figure 7**).

EIF4A3 could bind to intronic sequences surrounding circularized exons to facilitate or suppress circularization, leading to the changes of circRNAs expression. For example, EIF4A3 could bind to the flanking sequences of circ_0084615, a circRNA derived from aspartate beta-hydroxylase (ASPH), through the potential binding sites, and positively regulated circ_0084615 expression. Circ_0084615 promoted CRC cell proliferation, migration, invasion and angiogenesis as well as tumor growth in murine xenograft model via miR-599/ONECUT2 pathway, which increased the expression of ZEB2, Vimentin and VEGFA, but decreased E-cadherin expression (59). By binding to flanking sequences, EIF4A3 suppressed the cyclization of circPTEN1 and caused the decrease of circPTEN1 expression. CircPTEN1 was frequently downregulated in CRC and low expression of circPTEN1 predicted poor survival. And circPTEN1 suppressed CRC metastasis via inhibition of TGF-β/Smad-mediated EMT (60) (**Figure 7**).

In addition, cancer-associated fibroblasts (CAF) secreted exosomes cricN4BP2L2 could bind to EIF4A3 and positively regulate its expression, and subsequently caused the activation of PI3K/AKT/mTOR axis in CRC cells, which promoted CRC cells stemness and oxaliplatin resistance (61) (**Figure 7**).

#### 4.1.11 DDX54

DDX54 was overexpressed in CRC tissues, and high level of DDX54 was associated with tumor stage and distant metastasis as well as shorter overall survival in patients with CRC. DDX54 knockdown significantly inhibited tumor growth in subcutaneously injected nude mice (67). *In vitro*, DDX54 was demonstrated to promote the proliferation, migration, invasion and EMT of CRC cells through activating p65 and AKT pathway (67).

#### 4.1.12 DDX56

DDX56 expression was upregulated in CRC tumor cells and mainly localized to their cytoplasm. Its upregulation was positively correlated with the amplification of DDX56. High DDX56 expression was correlated with lymphatic invasion and distant metastasis and was an independent poor prognostic factor in CRC patients (71). Knockdown of DDX56 inhibited CRC cell proliferation and suspended cell cycle progression from G2/M to G1 phase. Consistently, downregulation of DDX56 suppressed tumor growth in a xenograft model (71). Mechanismly, DDX56 promoted CRC cell proliferation by inducing alternative splicing of WEE1, which was a crucial component of the G2/M cell cycle checkpoint and played a tumor-suppressing role in CRC, leading to the reduced expression of WEE1 (71).

### 4.2 DDX60 seems to function as a tumor-suppressing factor and is a potential target for CRC immunotherapy

DDX60 expression was downregulated in CRC cancerous tissues compared with normal tissues. And DDX60 expression was positively related to better survival probability of CRC patients. CRC tumors with increased DDX60 showed more proinflammatory phenotypes, such as increased infiltration of dendritic cells, CD4^+^, and CD8^+^ T cells than that with low DDX60 tumors (77). In CRC cells, DDX60 could positively regulate MHC-I expression, which was associated with responders in anti-PD1 immunotherapy and was required for T cell surveillance in CRC (77). This suggested that DDX60 might have an important clinical significance in CRC and could serve as a potential immune therapeutic target. However, the exact molecular mechanism of DDX60 regulating MHC-I was dimness.

### 4.3 Dual role of DEAD-box proteins in colorectal cancer

Strikingly, a portion of the DEAD-box proteins have controversial roles in CRC. For instance, DDX3X and DDX58 could function as both tumor-promoting and tumor-suppressing roles based on different molecular pathways. Hence, elucidating the dual role of these DEAD-box proteins can better understand their functions in CRC.

#### 4.3.1 DDX3X

**Figure 8:**
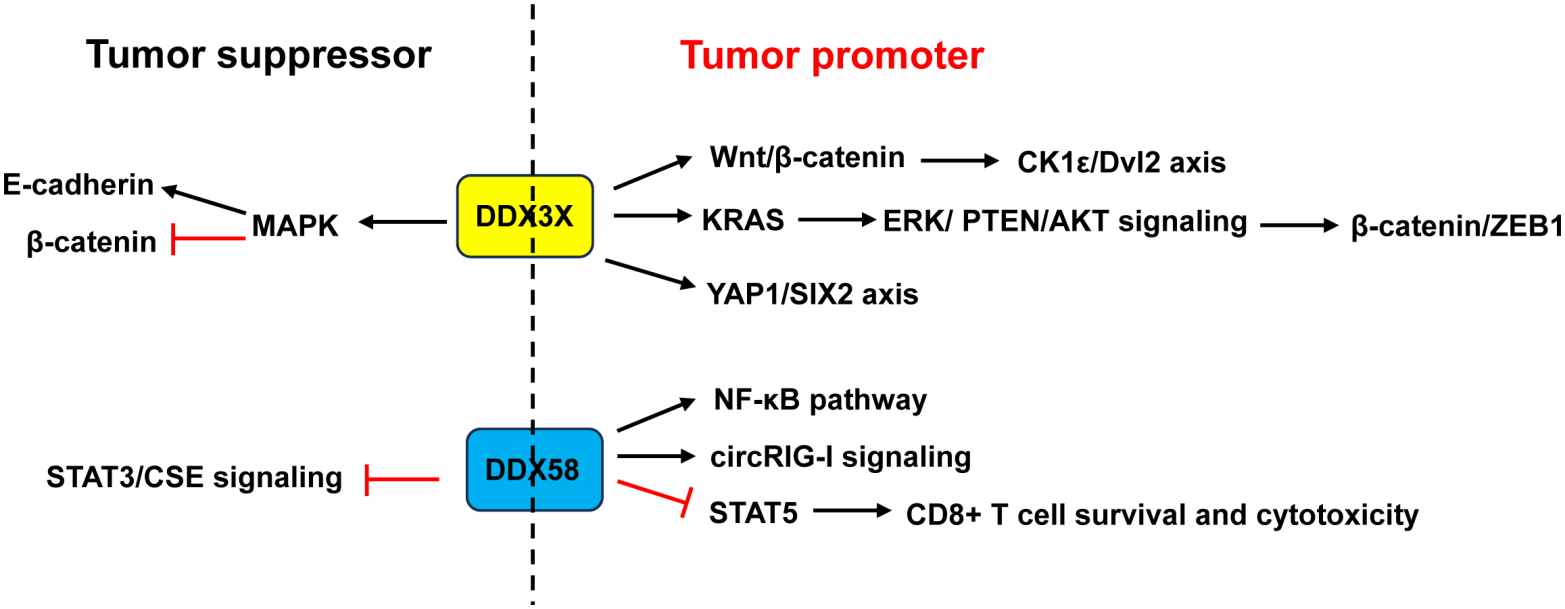
Dual role of DDX3X and DDX58 in CRC. Studies demonstrated that DDX3X acted as a tumor promoter in CRC through activating the Wnt/β-catenin/CK1ϵ/Dvl2 pathway and YAP1/SIX2 axis, or enhancing KRAS transcription and subsequently activating the β-catenin/ZEB1 axis via the ERK/PTEN/AKT signaling pathway. Other studies revealed that DDX3X acted as a tumor suppressor by inhibiting E-cadherin and activating β-catenin signaling via suppressing the MAPK pathway. About DDX58, some studies showed that it acted as a tumor promoter in CRC through activation of the NF-κB pathway and circRIG-I signaling, or through restraining CD8^+^ T cell survival and cytotoxicity by restricting STAT5 activation. Other study found that DDX58 functioned as a tumor suppressor by interacting with STAT3 and inhibiting the STAT3/CSE signaling. ZEB1, zinc finger E-box binding homeobox 1.

Most papers indicated that DDX3X acted as a tumor promoter in CRC (**Figure 8**). DDX3X knockdown or inhibition of DDX3X with the small molecule inhibitor RK-33 in CRC cell lines reduced proliferation and caused a G1 arrest via inhibition of the Wnt signaling (79). He et al. (80) revealed that DDX3X promoted the invasion capability of CRC cells and xenograft lung tumor nodules formation via the CK1ϵ/Dvl2 axis due to activation of the Wnt/β-catenin signaling. Moreover, DDX3X enhanced oncogenic KRAS transcription via an increase in SP1 binding to its promoter and subsequently activated the β-catenin/zinc finger E-box binding homeobox 1 (ZEB1) axis via the ERK/PTEN/AKT signaling pathway (138). DDX3 not only promoted cell invasion in KRAS-mutated CRC cells (138), but also increased tumor aggressiveness and cetuximab resistance in KRAS-wild-type CRC, which was regulated by the YAP1/SIX2 axis (139). Nuclear DDX3X that was partially mediated by nuclear exporter chromosome region maintenance 1 (CRM1) depending on the N-terminal nuclear export signal predicted worse survival in CRC patients (99). Avenanthramide A, the effective ingredient in avenanthramides, exhibited anti-cancer activities in CRC cells with DDX3X highly expressed via directly binding to ATP-binding domain of oncogenic protein DDX3X to block the ATPase activity of DDX3X and induce its degradation (140). USP7, a deubiquitinase, maintained the EMT state in CRC via stabilizing DDX3X and augmenting Wnt/β-catenin signaling (141).

However, Su et al. (81) and Shen et al. (82) argued that CRC patients with low DDX3X expression had poor prognosis and metastasis. Downregulation of DDX3X enhanced proliferation, migration and invasion of CRC cells *in vitro* and enhanced tumor metastasis *in vivo* by inhibiting E-cadherin and activating β-catenin signaling via suppressing the MAPK pathway (81, 82) (**Figure 8**).

#### 4.3.2 DDX58/RIG-I

DDX58 is an important immune-related gene that is closely associated with immune cell infiltration and tumor prognosis (142). Liu et al. (85) demonstrated that RIG-I promoted CRC cell proliferation and glucose metabolism, and suppressed apoptosis by activation of the NF-κB signaling pathway. Frameshift germline mutations of RIG-I was found in CRC patients. Mutant RIG-I could enhance inflammatory response that contributed to CRC development through activation of circRIG-I signaling (143). Jiang and his coworkers found that intrinsic RIG-I antagonized CD8^+^ T cell survival and effector function in tumor microenvironment and attenuated CD8^+^ T cell-mediated antitumor immunity (86). Mechanistically, RIG-I restrained CD8^+^ T cell survival and cytotoxicity by restricting STAT5 activation via competitive sequestering of HSP90 (86). The frequency of RIG-I^+^ tumor-infiltrating CD8^+^ T cells was positively correlated with compromised CD8^+^ T cells’ survival and cytotoxicity as well as poor prognosis in CRC patients (86) (**Figure 8**).

In contrast, Deng et al. (84) demonstrated that CRC patients with a low expression of DDX58 had a lower 5-year survival rate. Overexpression of DDX58 or its agonist SB9200 suppressed the migration and invasion of CRC cells as well as tumor growth in a nude mouse xenograft model. Mechanistically, the anti-cancer effects of DDX58 were achieved by interacting with STAT3 and inhibiting the STAT3/CSE signaling (84) (**Figure 8**).

## 5 Conclusions and future perspectives

Although the DEAD-box helicases have conserved helicase core, they perform different cellular functions and act via diverse mechanisms, which may be related to their variable auxiliary domains, cellular localization, and binding partners. Growing evidence has found that the DEAD-box helicases perform an crucial role in CRC. Current studies have demonstrated that most of these proteins, such as DDX1, DDX2, DDX5, DDX17, DDX6, DDX10, DDX21, DDX27, DDX39B, DDX46, EIF4A3, DDX54, DDX56, are upregulated and have tumor-promoting roles in CRC. DDX60 is downregulated in CRC tissues and lower levels of DDX60 are associated with poor prognosis in patients. DDX60 seems to play tumor-suppressing role in CRC via modulating tumor microenvironment; however, its exact function and mechanism need further study. It’s interesting that some DEAD-box helicases, including DDX3X and DDX58, exhibit opposite effects on CRC. Current studies revealed that they employ different mechanism during the divergent function, however, it is still confusing whether the controversial roles result from experimental limitations or other undiscovered mechanisms.

What’s more, the DEAD-box helicases also play vital roles in CRC treatment. For example, EIF4A2 inhibitor improved oxaliplatin sensitivity (17). DDX27 knockdown promoted chemosensitivity in CRC (44, 45). The upregulation of EIF4A3 promoted oxaliplatin resistance in CRC (61). Currently, some compounds targeting the DEAD-box helicases have been developed (reviewed in (87)). For example, RX-5902 is a first-in-class anticancer compound targeting phosphorylated DDX5, leading to a large diminishment of nuclear β-catenin in murine triple-negative breast cancer models (87). Silvestrol is a specific inhibitor of the eIF4A. It inhibits eIF4A through increasing the affinity of eIF4A to its target mRNA to stall eIF4A on its RNA substrate. Silvestrol exhibits significant anticancer activity in breast and prostate cancer xenograft models (144). Hence, further development of novel DDX inhibitors and exploring their safety are needed to conquer CRC in future. Furthermore, CRC cells with DDX21 overexpression are highly sensitive to chemotherapeutic drugs due to the genome instability caused by DDX21 (37). It is necessary to evaluate whether DDX21 as well as other DEAD-box helicases can provide precision therapeutic strategies for CRC in clinic.

## Author contributions

BZ: Conceptualization, Project administration, Supervision, Writing – original draft. XC: Writing – original draft. QL: Writing – original draft. QC: Writing – original draft. SY: Supervision, Writing – review & editing.

## References

[1] Sung H Ferlay J Siegel RL Laversanne M Soerjomataram I Jemal A. Global cancer statistics 2020: GLOBOCAN estimates of incidence and mortality worldwide for 36 cancers in 185 countries. CA Cancer J Clin (2021) 71(3):209–49. doi: 10.3322/caac.21660 33538338

[2] Siegel RL Wagle NS Cercek A Smith RA Jemal A. Colorectal cancer statistics, 2023. CA Cancer J Clin (2023) 73(3):233–54. doi: 10.3322/caac.21772 36856579

[3] Dekker E Tanis PJ Vleugels JLA Kasi PM Wallace MB. Colorectal cancer. Lancet (2019) 394(10207):1467–80. doi: 10.1016/S0140-6736(19)32319-0 31631858

[4] Brenner H Kloor M Pox CP. Colorectal cancer. Lancet (2014) 383(9927):1490–502. doi: 10.1016/S0140-6736(13)61649-9 24225001

[5] Johdi NA Sukor NF. Colorectal cancer immunotherapy: options and strategies. Front Immunol (2020) 11:1624. doi: 10.3389/fimmu.2020.01624 33042104 PMC7530194

[6] Fairman-Williams ME Guenther UP Jankowsky E. SF1 and SF2 helicases: family matters. Curr Opin Struct Biol (2010) 20(3):313–24. doi: 10.1016/j.sbi.2010.03.011 PMC2916977 20456941

[7] Ali MAM. The DEAD-box protein family of RNA helicases: sentinels for a myriad of cellular functions with emerging roles in tumorigenesis. Int J Clin Oncol (2021) 26(5):795–825. doi: 10.1007/s10147-021-01892-1 33656655

[8] Gao H Wei H Yang Y Li H Liang J Ye J. Phase separation of DDX21 promotes colorectal cancer metastasis via MCM5-dependent EMT pathway. Oncogene (2023) 42(21):1704–15. doi: 10.1038/s41388-023-02687-6 PMC10202810 37029300

[9] Li L Germain DR Poon HY Hildebrandt MR Monckton EA McDonald D. DEAD box 1 facilitates removal of RNA and homologous recombination at DNA double-strand breaks. Mol Cell Biol (2016) 36(22):2794–810. doi: 10.1128/MCB.00415-16 PMC5086521 27550810

[10] Ullah R Li J Fang P Xiao S Fang L. DEAD/H-box helicases:Anti-viral and pro-viral roles during infections. Virus Res (2022) 309:198658. doi: 10.1016/j.virusres.2021.198658 34929216

[11] Tanaka K Ikeda N Miyashita K Nuriya H Hara T. DEAD box protein DDX1 promotes colorectal tumorigenesis through transcriptional activation of the LGR5 gene. Cancer Sci (2018) 109(8):2479–89. doi: 10.1111/cas.13661 PMC6113447 29869821

[12] Han K Wang FW Cao CH Ling H Chen JW Chen RX. CircLONP2 enhances colorectal carcinoma invasion and metastasis through modulating the maturation and exosomal dissemination of microRNA-17. Mol Cancer (2020) 19(1):60. doi: 10.1186/s12943-020-01184-8 32188489 PMC7079398

[13] Xue C Gu X Li G Bao Z Li L. Expression and functional roles of eukaryotic initiation factor 4A family proteins in human cancers. Front Cell Dev Biol (2021) 9:711965. doi: 10.3389/fcell.2021.711965 34869305 PMC8640450

[14] Li W Chen A Xiong L Chen T Tao F Lu Y. miR-133a acts as a tumor suppressor in colorectal cancer by targeting eIF4A1. Tumour Biol (2017) 39(5):1010428317698389. doi: 10.1177/1010428317698389 28466778

[15] Yang T Chen WC Shi PC Liu MR Jiang T Song H. Long noncoding RNA MAPKAPK5-AS1 promotes colorectal cancer progression by cis-regulating the nearby gene MK5 and acting as a let-7f-1-3p sponge. J Exp Clin Cancer Res (2020) 39(1):139. doi: 10.1158/1557-3265.LiqBiop20-B13 32690100 PMC7370515

[16] Meijer HA Kong YW Lu WT Wilczynska A Spriggs RV Robinson SW. Translational repression and eIF4A2 activity are critical for microRNA-mediated gene regulation. Science (2013) 340(6128):82–5. doi: 10.1126/science.1231197 23559250

[17] Chen ZH Qi JJ Wu QN Lu JH Liu ZX Wang Y. Eukaryotic initiation factor 4A2 promotes experimental metastasis and oxaliplatin resistance in colorectal cancer. J Exp Clin Cancer Res (2019) 38(1):196. doi: 10.1007/s00432-018-2796-0 31088567 PMC6518650

[18] Xu K Sun S Yan M Cui J Yang Y Li W. DDX5 and DDX17-multifaceted proteins in the regulation of tumorigenesis and tumor progression. Front Oncol (2022) 12:943032. doi: 10.3389/fonc.2022.943032 35992805 PMC9382309

[19] Sarkar M Khare V Guturi KK Das N Ghosh MK. The DEAD box protein p68: a crucial regulator of AKT/FOXO3a signaling axis in oncogenesis. Oncogene (2015) 34(47):5843–56. doi: 10.1038/onc.2015.42 25745998

[20] Kal S Chakraborty S Karmakar S Ghosh MK. Wnt/beta-catenin signaling and p68 conjointly regulate CHIP in colorectal carcinoma. Biochim Biophys Acta Mol Cell Res (2022) 1869(3):119185. doi: 10.1016/j.bbamcr.2021.119185 34890713

[21] Zou S Qin B Yang Z Wang W Zhang J Zhang Y. CSN6 mediates nucleotide metabolism to promote tumor development and chemoresistance in colorectal cancer. Cancer Res (2023) 83(3):414–27. doi: 10.1158/0008-5472.CAN-22-2145 36512632

[22] Shin S Rossow KL Grande JP Janknecht R. Involvement of RNA helicases p68 and p72 in colon cancer. Cancer Res (2007) 67(16):7572–8. doi: 10.1158/0008-5472.CAN-06-4652 17699760

[23] Zhao G Wang Q Zhang Y Gu R Liu M Li Q. DDX17 induces epithelial-mesenchymal transition and metastasis through the miR-149-3p/CYBRD1 pathway in colorectal cancer. Cell Death Dis (2023) 14(1):1. doi: 10.1038/s41419-022-05508-y 36593242 PMC9807641

[24] Iio A Takagi T Miki K Naoe T Nakayama A Akao Y. DDX6 post-transcriptionally down-regulates miR-143/145 expression through host gene NCR143/145 in cancer cells. Biochim Biophys Acta (2013) 1829(10):1102–10. doi: 10.1016/j.bbagrm.2013.07.010 23932921

[25] Smillie DA Sommerville J. RNA helicase p54 (DDX6) is a shuttling protein involved in nuclear assembly of stored mRNP particles. J Cell Sci (2002) 115(Pt 2):395–407. doi: 10.1242/jcs.115.2.395 11839790

[26] Kawahara C Yokota S Fujita H. DDX6 localizes to nuage structures and the annulus of mammalian spermatogenic cells. Histochem Cell Biol (2014) 141(1):111–21. doi: 10.1007/s00418-013-1153-2 24141902

[27] Lin F Wang R Shen JJ Wang X Gao P Dong K. Knockdown of RCK/p54 expression by RNAi inhibits proliferation of human colorectal cancer cells in vitro and in vivo. Cancer Biol Ther (2008) 7(10):1669–76. doi: 10.4161/cbt.7.10.6660 18769115

[28] Taniguchi K Sugito N Kumazaki M Shinohara H Yamada N Matsuhashi N. Positive feedback of DDX6/c-Myc/PTB1 regulated by miR-124 contributes to maintenance of the Warburg effect in colon cancer cells. Biochim Biophys Acta (2015) 1852(9):1971–80. doi: 10.1016/j.bbadis.2015.06.022 26144048

[29] Soltanieh S Osheim YN Spasov K Trahan C Beyer AL Dragon F. DEAD-box RNA helicase Dbp4 is required for small-subunit processome formation and function. Mol Cell Biol (2015) 35(5):816–30. doi: 10.1128/MCB.01348-14 PMC4323488 25535329

[30] Popova B Wang D Patz C Akkermann D Lazaro DF Galka D. DEAD-box RNA helicase Dbp4/DDX10 is an enhancer of alpha-synuclein toxicity and oligomerization. PloS Genet (2021) 17(3):e1009407. doi: 10.1371/journal.pgen.1009407 33657088 PMC7928443

[31] Yassin ER Abdul-Nabi AM Takeda A Yaseen NR. Effects of the NUP98-DDX10 oncogene on primary human CD34+ cells: role of a conserved helicase motif. Leukemia (2010) 24(5):1001–11. doi: 10.1038/leu.2010.42 PMC2868946 20339440

[32] Zhou X Liu Z He T Zhang C Jiang M Jin Y. DDX10 promotes the proliferation and metastasis of colorectal cancer cells via splicing RPL35. Cancer Cell Int (2022) 22(1):58. doi: 10.1186/s12935-022-02478-1 35109823 PMC8812018

[33] Kim DS Camacho CV Nagari A Malladi VS Challa S Kraus WL. Activation of PARP-1 by snoRNAs Controls Ribosome Biogenesis and Cell Growth via the RNA Helicase DDX21. Mol Cell (2019) 75(6):1270–1285 e14. doi: 10.1016/j.molcel.2019.06.020 31351877 PMC6754283

[34] Miao W Porter DF Lopez-Pajares V Siprashvili Z Meyers RM Bai Y. Glucose dissociates DDX21 dimers to regulate mRNA splicing and tissue differentiation. Cell (2023) 186(1):80–97 e26. doi: 10.1016/j.cell.2022.12.004 36608661 PMC10171372

[35] Wu W Qu Y Yu S Wang S Yin Y Liu Q. Caspase-dependent cleavage of DDX21 suppresses host innate immunity. mBio (2021) 12(3):e0100521. doi: 10.1128/mBio.01005-21 34125604 PMC8262918

[36] Li J Fang P Zhou Y Wang D Fang L Xiao S. DEAD-box RNA helicase 21 negatively regulates cytosolic RNA-mediated innate immune signaling. Front Immunol (2022) 13:956794. doi: 10.3389/fimmu.2022.956794 36032158 PMC9399600

[37] Xie J Wen M Zhang J Wang Z Wang M Qiu Y. The roles of RNA helicases in DNA damage repair and tumorigenesis reveal precision therapeutic strategies. Cancer Res (2022) 82(5):872–84. doi: 10.1158/0008-5472.CAN-21-2187 34987058

[38] Wang K Li B Fan P Ren X Jiang H. Downregulation of DEAD-box helicase 21 (DDX21) inhibits proliferation, cell cycle, and tumor growth in colorectal cancer via targeting cell division cycle 5-like (CDC5L). Bioengineered (2021) 12(2):12647–58. doi: 10.1080/21655979.2021.2011636 PMC8810101 34903139

[39] Lu P Yu Z Wang K Zhai Y Chen B Liu M. DDX21 interacts with WDR5 to promote colorectal cancer cell proliferation by activating CDK1 expression. J Cancer (2022) 13(5):1530–9. doi: 10.7150/jca.69216 PMC8965128 35371306

[40] Kellner M Rohrmoser M Forne I Voss K Burger K Muhl B. DEAD-box helicase DDX27 regulates 3’ end formation of ribosomal 47S RNA and stably associates with the PeBoW-complex. Exp Cell Res (2015) 334(1):146–59. doi: 10.1016/j.yexcr.2015.03.017 25825154

[41] Bennett AH O'Donohue MF Gundry SR Chan AT Widrick J Draper I. RNA helicase, DDX27 regulates skeletal muscle growth and regeneration by modulation of translational processes. PloS Genet (2018) 14(3):e1007226. doi: 10.1371/journal.pgen.1007226 29518074 PMC5843160

[42] Xiaoqian W Bing Z Yangwei L Yafei Z Tingting Z Yi W. DEAD-box helicase 27 promotes hepatocellular carcinoma progression through ERK signaling. Technol Cancer Res Treat (2021) 20:15330338211055953. doi: 10.1177/15330338211055953 34855554 PMC8649435

[43] Tang J Chen H Wong CC Liu D Li T Wang X. DEAD-box helicase 27 promotes colorectal cancer growth and metastasis and predicts poor survival in CRC patients. Oncogene (2018) 37(22):3006–21. doi: 10.1038/s41388-018-0196-1 PMC5978808 29535419

[44] Yang C Li D Bai Y Song S Yan P Wu R. DEAD-box helicase 27 plays a tumor-promoter role by regulating the stem cell-like activity of human colorectal cancer cells. Onco Targets Ther (2019) 12:233–41. doi: 10.2147/OTT.S190814 PMC6314319 30643421

[45] Guo Y Hu G Tian B Ma M Long F Chen M. Circ_RNF13 regulates the stemness and chemosensitivity of colorectal cancer by transcriptional regulation of DDX27 mediated by TRIM24 stabilization. Cancers (Basel) (2022) 14(24):6218. doi: 10.3390/cancers14246218 36551703 PMC9776557

[46] Thomas M Lischka P Muller R Stamminger T. The cellular DExD/H-box RNA-helicases UAP56 and URH49 exhibit a CRM1-independent nucleocytoplasmic shuttling activity. PloS One (2011) 6(7):e22671. doi: 10.1371/journal.pone.0022671 21799930 PMC3142171

[47] Yoo HH Chung IK. Requirement of DDX39 DEAD box RNA helicase for genome integrity and telomere protection. Aging Cell (2011) 10(4):557–71. doi: 10.1111/j.1474-9726.2011.00696.x 21388492

[48] Awasthi S Chakrapani B Mahesh A Chavali PL Chavali S Dhayalan A. DDX39B promotes translation through regulation of pre-ribosomal RNA levels. RNA Biol (2018) 15(9):1157–66. doi: 10.1080/15476286.2018.1517011 PMC6284572 30176153

[49] Wisskirchen C Ludersdorfer TH Muller DA Moritz E Pavlovic J. Interferon-induced antiviral protein MxA interacts with the cellular RNA helicases UAP56 and URH49. J Biol Chem (2011) 286(40):34743–51. doi: 10.1074/jbc.M111.251843 PMC3186362 21859714

[50] He C Li A Lai Q Ding J Yan Q Liu S. The DDX39B/FUT3/TGFbetaR-I axis promotes tumor metastasis and EMT in colorectal cancer. Cell Death Dis (2021) 12(1):74. doi: 10.1038/s41419-020-03360-6 33436563 PMC7803960

[51] Zhao G Yuan H Li Q Zhang J Guo Y Feng T. DDX39B drives colorectal cancer progression by promoting the stability and nuclear translocation of PKM2. Signal Transduct Target Ther (2022) 7(1):275. doi: 10.1038/s41392-022-01096-7 35973989 PMC9381590

[52] Zhang Z Rigo N Dybkov O Fourmann JB Will CL Kumar V. Structural insights into how Prp5 proofreads the pre-mRNA branch site. Nature (2021) 596(7871):296–300. doi: 10.1038/s41586-021-03789-5 34349264 PMC8357632

[53] Zheng Q Hou J Zhou Y Li Z Cao X. The RNA helicase DDX46 inhibits innate immunity by entrapping m(6)A-demethylated antiviral transcripts in the nucleus. Nat Immunol (2017) 18(10):1094–103. doi: 10.1038/ni.3830 28846086

[54] Li M Ma Y Huang P Du A Yang X Zhang S. Lentiviral DDX46 knockdown inhibits growth and induces apoptosis in human colorectal cancer cells. Gene (2015) 560(2):237–44. doi: 10.1016/j.gene.2015.02.020 25680556

[55] Ye J She X Liu Z He Z Gao X Lu L. Eukaryotic initiation factor 4A-3: A review of its physiological role and involvement in oncogenesis. Front Oncol (2021) 11:712045. doi: 10.3389/fonc.2021.712045 34458150 PMC8386015

[56] Sakellariou D Frankel LB. EIF4A3: a gatekeeper of autophagy. Autophagy (2021) 17(12):4504–5. doi: 10.1080/15548627.2021.1985881 PMC8726629 34643458

[57] Choe J Ryu I Park OH Park J Cho H Yoo JS. eIF4AIII enhances translation of nuclear cap-binding complex-bound mRNAs by promoting disruption of secondary structures in 5’UTR. Proc Natl Acad Sci U.S.A. (2014) 111(43):E4577–86. doi: 10.1073/pnas.1409695111 PMC4217471 25313076

[58] Wang X Liu S Xu B Liu Y Kong P Li C. circ-SIRT1 promotes colorectal cancer proliferation and EMT by recruiting and binding to eIF4A3. Anal Cell Pathol (Amst) (2021) 2021:5739769. doi: 10.1155/2021/5739769 34660182 PMC8519704

[59] Jiang Z Tai Q Xie X Hou Z Liu W Yu Z. EIF4A3-induced circ_0084615 contributes to the progression of colorectal cancer via miR-599/ONECUT2 pathway. J Exp Clin Cancer Res (2021) 40(1):227. doi: 10.1186/s13046-021-02029-y 34253241 PMC8273970

[60] Zheng L Liang H Zhang Q Shen Z Sun Y Zhao X. circPTEN1, a circular RNA generated from PTEN, suppresses cancer progression through inhibition of TGF-beta/Smad signaling. Mol Cancer (2022) 21(1):41. doi: 10.1186/s12943-022-01495-y 35135542 PMC8822707

[61] Qu Z Yang KD Luo BH Zhang F. CAFs-secreted exosomal cricN4BP2L2 promoted colorectal cancer stemness and chemoresistance by interacting with EIF4A3. Exp Cell Res (2022) 418(2):113266. doi: 10.1016/j.yexcr.2022.113266 35752345

[62] Kanno Y Serikawa T Inajima J Inouye Y. DP97, a DEAD box DNA/RNA helicase, is a target gene-selective co-regulator of the constitutive androstane receptor. Biochem Biophys Res Commun (2012) 426(1):38–42. doi: 10.1016/j.bbrc.2012.08.027 22910411

[63] Rajendran RR Nye AC Frasor J Balsara RD Martini PG Katzenellenbogen BS. Regulation of nuclear receptor transcriptional activity by a novel DEAD box RNA helicase (DP97). J Biol Chem (2003) 278(7):4628–38. doi: 10.1074/jbc.M210066200 12466272

[64] Milek M Imami K Mukherjee N Bortoli F Zinnall U Hazapis O. DDX54 regulates transcriptome dynamics during DNA damage response. Genome Res (2017) 27(8):1344–59. doi: 10.1101/gr.218438.116 PMC5538551 28596291

[65] Liu P Zhang Y Li X Ma M. DEAD-box helicase 54 regulates microglial inflammatory response in rats with chronic constriction injuries through NF-kappaB/NLRP3 signaling axis. J Neurophysiol (2023) 130(2):392–400. doi: 10.1152/jn.00411.2022 37377223

[66] Jin G Zhang J Cao T Chen B Tian Y Shi Y. Exosome-mediated lncRNA SND1-IT1 from gastric cancer cells enhances Malignant transformation of gastric mucosa cells via up-regulating SNAIL1. J Transl Med (2022) 20(1):284. doi: 10.1186/s12967-022-03306-w 35739527 PMC9229915

[67] Yu Y Wang JL Meng LL Hu CT Yan ZW He ZP. DDX54 plays a cancerous role through activating P65 and AKT signaling pathway in colorectal cancer. Front Oncol (2021) 11:650360. doi: 10.3389/fonc.2021.650360 33968751 PMC8097168

[68] Taschuk F Tapescu I Moy RH Cherry S. DDX56 binds to chikungunya virus RNA to control infection. mBio (2020) 11(5):e02623-20. doi: 10.1128/mBio.02623-20 33109765 PMC7593974

[69] Wang J Liu J Ye M Liu F Wu S Huang J. Ddx56 maintains proliferation of mouse embryonic stem cells via ribosome assembly and interaction with the Oct4/Sox2 complex. Stem Cell Res Ther (2020) 11(1):314. doi: 10.1186/s13287-020-01800-w 32703285 PMC7376950

[70] Zhou H Du Y Wei X Song C Song J Xu N. DDX56 transcriptionally activates MIST1 to facilitate tumorigenesis of HCC through PTEN-AKT signaling. Theranostics (2022) 12(14):6069–87. doi: 10.7150/thno.72471 PMC9475456 36168636

[71] Kouyama Y Masuda T Fujii A Ogawa Y Sato K Tobo T. Oncogenic splicing abnormalities induced by DEAD-Box Helicase 56 amplification in colorectal cancer. Cancer Sci (2019) 110(10):3132–44. doi: 10.1111/cas.14163 PMC6778637 31390121

[72] Wu Q Luo X Terp MG Li Q Li Y Shen L. DDX56 modulates post-transcriptional Wnt signaling through miRNAs and is associated with early recurrence in squamous cell lung carcinoma. Mol Cancer (2021) 20(1):108. doi: 10.1186/s12943-021-01403-w 34446021 PMC8393456

[73] Xie J Li X Yang S Yan Z Chen L Yang Y. DDX56 inhibits PRV replication through regulation of IFN-beta signaling pathway by targeting cGAS. Front Microbiol (2022) 13:932842. doi: 10.3389/fmicb.2022.932842 36090064 PMC9450509

[74] Oshiumi H Miyashita M Okamoto M Morioka Y Okabe M Matsumoto M. DDX60 is involved in RIG-I-dependent and independent antiviral responses, and its function is attenuated by virus-induced EGFR activation. Cell Rep (2015) 11(8):1193–207. doi: 10.1016/j.celrep.2015.04.047 25981042

[75] Sadic M Schneider WM Katsara O Medina GN Fisher A Mogulothu A. DDX60 selectively reduces translation off viral type II internal ribosome entry sites. EMBO Rep (2022) 23(12):e55218. doi: 10.15252/embr.202255218 36256515 PMC9724679

[76] Lai T Su X Chen E Tao Y Zhang S Wang L. The DEAD-box RNA helicase, DDX60, Suppresses immunotherapy and promotes Malignant progression of pancreatic cancer. Biochem Biophys Rep (2023) 34:101488. doi: 10.1016/j.bbrep.2023.101488 37274827 PMC10236181

[77] Geng N Hu T He C. Identification of DDX60 as a regulator of MHC-I class molecules in colorectal cancer. Biomedicines (2022) 10(12):3092. doi: 10.3390/biomedicines10123092 36551849 PMC9775109

[78] Mo J Liang H Su C Li P Chen J Zhang B. DDX3X: structure, physiologic functions and cancer. Mol Cancer (2021) 20(1):38. doi: 10.1186/s12943-021-01325-7 33627125 PMC7903766

[79] Heerma van Voss MR Vesuna F Trumpi K Brilliant J Berlinicke C de Leng W. Identification of the DEAD box RNA helicase DDX3 as a therapeutic target in colorectal cancer. Oncotarget (2015) 6(29):28312–26. doi: 10.18632/oncotarget.4873 PMC4695062 26311743

[80] He TY Wu DW Lin PL Wang L Huang CC Chou MC. DDX3 promotes tumor invasion in colorectal cancer via the CK1epsilon/Dvl2 axis. Sci Rep (2016) 6:21483. doi: 10.1038/srep21483 26892600 PMC4759588

[81] Su CY Lin TC Lin YF Chen MH Lee CH Wang HY. DDX3 as a strongest prognosis marker and its downregulation promotes metastasis in colorectal cancer. Oncotarget (2015) 6(21):18602–12. doi: 10.18632/oncotarget.4329 PMC4621913 26087195

[82] Shen L Zhang J Xu M Zheng Y Wang M Yang S. DDX3 acts as a tumor suppressor in colorectal cancer as loss of DDX3 in advanced cancer promotes tumor progression by activating the MAPK pathway. Int J Biol Sci (2022) 18(10):3918–33. doi: 10.7150/ijbs.73491 PMC9274493 35844798

[83] Sun H Yang Z Teng Z Zhang Y Han Z Xu C. DDX58 expression promotes inflammation and growth arrest in Sertoli cells by stabilizing p65 mRNA in patients with Sertoli cell-only syndrome. Front Immunol (2023) 14:1135753. doi: 10.3389/fimmu.2023.1135753 37033952 PMC10073560

[84] Deng Y Fu H Han X Li Y Zhao W Zhao X. Activation of DDX58/RIG−I suppresses the growth of tumor cells by inhibiting STAT3/CSE signaling in colon cancer. Int J Oncol (2022) 61(4):120. doi: 10.3892/ijo.2022.5410 36004488 PMC9450811

[85] Liu Y Ye S Zhu Y Chen L Zhang Z. RIG-I promotes cell viability, colony formation, and glucose metabolism and inhibits cell apoptosis in colorectal cancer by NF-kappaB signaling pathway. Dis Markers (2022) 2022:1247007. doi: 10.1155/2022/1247007 35242239 PMC8888050

[86] Jiang X Lin J Shangguan C Wang X Xiang B Chen J. Intrinsic RIG-I restrains STAT5 activation to modulate antitumor activity of CD8+ T cells. J Clin Invest (2023) 133(9):e160790. doi: 10.1172/JCI160790 36927693 PMC10145944

[87] Zhang L Li X. DEAD-box RNA helicases in cell cycle control and clinical therapy. Cells (2021) 10(6):1540. doi: 10.3390/cells10061540 34207140 PMC8234093

[88] Ali MAM. DEAD-box RNA helicases: The driving forces behind RNA metabolism at the crossroad of viral replication and antiviral innate immunity. Virus Res (2021) 296:198352. doi: 10.1016/j.virusres.2021.198352 33640359

[89] Hilbert M Karow AR Klostermeier D. The mechanism of ATP-dependent RNA unwinding by DEAD box proteins. Biol Chem (2009) 390(12):1237–50. doi: 10.1515/BC.2009.135 19747077

[90] Cargill M Venkataraman R Lee S. DEAD-box RNA helicases and genome stability. Genes (Basel) (2021) 12(10):1471. doi: 10.3390/genes12101471 34680866 PMC8535883

[91] Abdullah SW Wu J Zhang Y Bai M Guan J Liu X. DDX21, a host restriction factor of FMDV IRES-dependent translation and replication. Viruses (2021) 13(9):1765. doi: 10.3390/v13091765 34578346 PMC8473184

[92] Xu L Khadijah S Fang S Wang L Tay FP Liu DX. The cellular RNA helicase DDX1 interacts with coronavirus nonstructural protein 14 and enhances viral replication. J Virol (2010) 84(17):8571–83. doi: 10.1128/JVI.00392-10 PMC2918985 20573827

[93] Germain DR Graham K Glubrecht DD Hugh JC Mackey JR Godbout R. DEAD box 1: a novel and independent prognostic marker for early recurrence in breast cancer. Breast Cancer Res Treat (2011) 127(1):53–63. doi: 10.1007/s10549-010-0943-7 20499159

[94] Lu WT Wilczynska A Smith E Bushell M. The diverse roles of the eIF4A family: you are the company you keep. Biochem Soc Trans (2014) 42(1):166–72. doi: 10.1042/BST20130161 24450646

[95] Ozes AR Feoktistova K Avanzino BC Fraser CS. Duplex unwinding and ATPase activities of the DEAD-box helicase eIF4A are coupled by eIF4G and eIF4B. J Mol Biol (2011) 412(4):674–87. doi: 10.1016/j.jmb.2011.08.004 PMC3175293 21840318

[96] Waldron JA Tack DC Ritchey LE Gillen SL Wilczynska A Turro E. mRNA structural elements immediately upstream of the start codon dictate dependence upon eIF4A helicase activity. Genome Biol (2019) 20(1):300. doi: 10.1186/s13059-019-1901-2 31888698 PMC6936103

[97] Modelska A Turro E Russell R Beaton J Sbarrato T Spriggs K. The Malignant phenotype in breast cancer is driven by eIF4A1-mediated changes in the translational landscape. Cell Death Dis (2015) 6(1):e1603. doi: 10.1038/cddis.2014.542 25611378 PMC4669741

[98] Lacroix M Beauchemin H Khandanpour C Moroy T. The RNA helicase DDX3 and its role in c-MYC driven germinal center-derived B-cell lymphoma. Front Oncol (2023) 13:1148936. doi: 10.3389/fonc.2023.1148936 37035206 PMC10081492

[99] Heerma van Voss MR Vesuna F Bol GM Meeldijk J Raman A Offerhaus GJ. Nuclear DDX3 expression predicts poor outcome in colorectal and breast cancer. Onco Targets Ther (2017) 10:3501–13. doi: 10.2147/OTT.S140639 PMC5522823 28761359

[100] Botlagunta M Vesuna F Mironchik Y Raman A Lisok A Winnard P Jr. Oncogenic role of DDX3 in breast cancer biogenesis. Oncogene (2008) 27(28):3912–22. doi: 10.1038/onc.2008.33 PMC5576029 18264132

[101] Samir P Kesavardhana S Patmore DM Gingras S Malireddi RKS Karki R. DDX3X acts as a live-or-die checkpoint in stressed cells by regulating NLRP3 inflammasome. Nature (2019) 573(7775):590–4. doi: 10.1038/s41586-019-1551-2 PMC6980284 31511697

[102] Ditton HJ Zimmer J Kamp C Rajpert-De Meyts E Vogt PH. The AZFa gene DBY (DDX3Y) is widely transcribed but the protein is limited to the male germ cells by translation control. Hum Mol Genet (2004) 13(19):2333–41. doi: 10.1093/hmg/ddh240 15294876

[103] Giraud G Terrone S Bourgeois CF. Functions of DEAD box RNA helicases DDX5 and DDX17 in chromatin organization and transcriptional regulation. BMB Rep (2018) 51(12):613–22. doi: 10.5483/BMBRep.2018.51.12.234 PMC6330936 30293550

[104] Kao SH Cheng WC Wang YT Wu HT Yeh HY Chen YJ. Regulation of miRNA biogenesis and histone modification by K63-polyubiquitinated DDX17 controls cancer stem-like features. Cancer Res (2019) 79(10):2549–63. doi: 10.1158/0008-5472.CAN-18-2376 30877109

[105] Di Stefano B Luo EC Haggerty C Aigner S Charlton J Brumbaugh J. The RNA helicase DDX6 controls cellular plasticity by modulating P-body homeostasis. Cell Stem Cell (2019) 25(5):622–638 e13. doi: 10.1016/j.stem.2019.08.018 31588046 PMC7247364

[106] Kim J Muraoka M Okada H Toyoda A Ajima R Saga Y. The RNA helicase DDX6 controls early mouse embryogenesis by repressing aberrant inhibition of BMP signaling through miRNA-mediated gene silencing. PloS Genet (2022) 18(10):e1009967. doi: 10.1371/journal.pgen.1009967 36197846 PMC9534413

[107] Marcon BH Rebelatto CK Cofre AR Dallagiovanna B Correa A. DDX6 helicase behavior and protein partners in human adipose tissue-derived stem cells during early adipogenesis and osteogenesis. Int J Mol Sci (2020) 21(7):2607. doi: 10.3390/ijms21072607 32283676 PMC7177724

[108] Shi JH Hao YJ. DDX10 overexpression predicts worse prognosis in osteosarcoma and its deletion prohibits cell activities modulated by MAPK pathway. Biochem Biophys Res Commun (2019) 510(4):525–9. doi: 10.1016/j.bbrc.2019.01.114 30738579

[109] Gai M Bo Q Qi L. Epigenetic down-regulated DDX10 promotes cell proliferation through Akt/NF-kappaB pathway in ovarian cancer. Biochem Biophys Res Commun (2016) 469(4):1000–5. doi: 10.1016/j.bbrc.2015.12.069 26713367

[110] Pallett MA Lu Y Smith GL. DDX50 is a viral restriction factor that enhances IRF3 activation. Viruses (2022) 14(2):316. doi: 10.3390/v14020316 35215908 PMC8875258

[111] Westermarck J Weiss C Saffrich R Kast J Musti AM Wessely M. The DEXD/H-box RNA helicase RHII/Gu is a co-factor for c-Jun-activated transcription. EMBO J (2002) 21(3):451–60. doi: 10.1093/emboj/21.3.451 PMC125820 11823437

[112] Han P Ye W Lv X Ma H Weng D Dong Y. DDX50 inhibits the replication of dengue virus 2 by upregulating IFN-beta production. Arch Virol (2017) 162(6):1487–94. doi: 10.1007/s00705-017-3250-3 28181036

[113] Shi P Guo Y Su Y Zhu M Fu Y Chi H. SUMOylation of DDX39A alters binding and export of antiviral transcripts to control innate immunity. J Immunol (2020) 205(1):168–80. doi: 10.4049/jimmunol.2000053 32393512

[114] Will CL Urlaub H Achsel T Gentzel M Wilm M Luhrmann R. Characterization of novel SF3b and 17S U2 snRNP proteins, including a human Prp5p homologue and an SF3b DEAD-box protein. EMBO J (2002) 21(18):4978–88. doi: 10.1093/emboj/cdf480 PMC126279 12234937

[115] Yang F Bian T Zhan X Chen Z Xing Z Larsen NA. Mechanisms of the RNA helicases DDX42 and DDX46 in human U2 snRNP assembly. Nat Commun (2023) 14(1):897. doi: 10.1038/s41467-023-36489-x 36797247 PMC9935549

[116] Bonaventure B Rebendenne A Chaves Valadao AL Arnaud-Arnould M Gracias S Garcia de Gracia F. The DEAD box RNA helicase DDX42 is an intrinsic inhibitor of positive-strand RNA viruses. EMBO Rep (2022) 23(11):e54061. doi: 10.15252/embr.202154061 36161446 PMC9638865

[117] Pirincal A Turan K. Human DDX56 protein interacts with influenza A virus NS1 protein and stimulates the virus replication. Genet Mol Biol (2021) 44(1):e20200158. doi: 10.1590/1678-4685-gmb-2020-0158 33749700 PMC7983190

[118] Zhang HX Liu ZX Sun YP Zhu J Lu SY Liu XS. Rig-I regulates NF-kappaB activity through binding to Nf-kappab1 3’-UTR mRNA. Proc Natl Acad Sci U.S.A. (2013) 110(16):6459–64. doi: 10.1073/pnas.1304432110 PMC3631665 23553835

[119] Soylemez Z Arikan ES Solak M Arikan Y Tokyol C Seker H. Investigation of the expression levels of CPEB4, APC, TRIP13, EIF2S3, EIF4A1, IFNg, PIK3CA and CTNNB1 genes in different stage colorectal tumors. Turk J Med Sci (2021) 51(2):661–74. doi: 10.3906/sag-2010-18 PMC8208508 33237662

[120] Chen H Liu H Qing G. Targeting oncogenic Myc as a strategy for cancer treatment. Signal Transduct Target Ther (2018) 3:5. doi: 10.1038/s41392-018-0008-7 29527331 PMC5837124

[121] Causevic M Hislop RG Kernohan NM Carey FA Kay RA Steele RJ. Overexpression and poly-ubiquitylation of the DEAD-box RNA helicase p68 in colorectal tumours. Oncogene (2001) 20(53):7734–43. doi: 10.1038/sj.onc.1204976 11753651

[122] Jacobs AM Nicol SM Hislop RG Jaffray EG Hay RT Fuller-Pace FV. SUMO modification of the DEAD box protein p68 modulates its transcriptional activity and promotes its interaction with HDAC1. Oncogene (2007) 26(40):5866–76. doi: 10.1038/sj.onc.1210387 17369852

[123] Wu N Jiang M Han Y Liu H Chu Y Liu H. O-GlcNAcylation promotes colorectal cancer progression by regulating protein stability and potential catcinogenic function of DDX5. J Cell Mol Med (2019) 23(2):1354–62. doi: 10.1111/jcmm.14038 PMC6349181 30484950

[124] Dai L Pan G Liu X Huang J Jiang Z Zhu X. High expression of ALDOA and DDX5 are associated with poor prognosis in human colorectal cancer. Cancer Manag Res (2018) 10:1799–806. doi: 10.2147/CMAR.S157925 PMC6029611 29988738

[125] Zhang M Weng W Zhang Q Wu Y Ni S Tan C. The lncRNA NEAT1 activates Wnt/beta-catenin signaling and promotes colorectal cancer progression via interacting with DDX5. J Hematol Oncol (2018) 11(1):113. doi: 10.1186/s13045-018-0656-7 30185232 PMC6125951

[126] Tabassum S Basu M Ghosh MK. The DEAD-box RNA helicase DDX5 (p68) and beta-catenin: The crucial regulators of FOXM1 gene expression in arbitrating colorectal cancer. Biochim Biophys Acta Gene Regul Mech (2023) 1866(2):194933. doi: 10.1016/j.bbagrm.2023.194933 36997114

[127] Liu Q Xin C Chen Y Yang J Chen Y Zhang W. PUM1 is overexpressed in colon cancer cells with acquired resistance to cetuximab. Front Cell Dev Biol (2021) 9:696558. doi: 10.3389/fcell.2021.696558 34447749 PMC8383298

[128] Wang X Yang P Zhang D Lu M Zhang C Sun Y. LncRNA SNHG14 promotes cell proliferation and invasion in colorectal cancer through modulating miR-519b-3p/DDX5 axis. J Cancer (2021) 12(16):4958–70. doi: 10.7150/jca.55495 PMC8247390 34234865

[129] Fu P Lin L Zhou H Zhao S Jie Z. Circular RNA circEGFR regulates tumor progression via the miR-106a-5p/DDX5 axis in colorectal cancer. Braz J Med Biol Res (2021) 54(8):e10940. doi: 10.1590/1414-431x2020e10940 34320120 PMC8302139

[130] Nakagawa Y Morikawa H Hirata I Shiozaki M Matsumoto A Maemura K. Overexpression of rck/p54, a DEAD box protein, in human colorectal tumours. Br J Cancer (1999) 80(5-6):914–7. doi: 10.1038/sj.bjc.6690441 PMC2362290 10360675

[131] Hashimoto K Nakagawa Y Morikawa H Niki M Egashira Y Hirata I. Co-overexpression of DEAD box protein rck/p54 and c-myc protein in human colorectal adenomas and the relevance of their expression in cultured cell lines. Carcinogenesis (2001) 22(12):1965–70. doi: 10.1093/carcin/22.12.1965 11751426

[132] Jung Y Lee S Choi HS Kim SN Lee E Shin Y. Clinical validation of colorectal cancer biomarkers identified from bioinformatics analysis of public expression data. Clin Cancer Res (2011) 17(4):700–9. doi: 10.1158/1078-0432.CCR-10-1300 21304002

[133] He L Wang F Tian H Xie Y Xie L Liu Z. The expression profile of RNA sensors in colorectal cancer and its correlation with cancer stages. Transl Cancer Res (2019) 8(4):1351–63. doi: 10.21037/tcr.2019.07.45 PMC8799165 35116878

[134] Tanaka A Wang JY Shia J Zhou Y Ogawa M Hendrickson RC. DEAD-box RNA helicase protein DDX21 as a prognosis marker for early stage colorectal cancer with microsatellite instability. Sci Rep (2020) 10(1):22085. doi: 10.1038/s41598-020-79049-9 33328538 PMC7745018

[135] Wang X Wu Z Qin W Sun T Lu S Li Y. Long non-coding RNA ZFAS1 promotes colorectal cancer tumorigenesis and development through DDX21-POLR1B regulatory axis. Aging (Albany NY) (2020) 12(22):22656–87. doi: 10.18632/aging.103875 PMC7746388 33202381

[136] Zhang H He C Guo X Fang Y Lai Q Wang X. DDX39B contributes to the proliferation of colorectal cancer through direct binding to CDK6/CCND1. Cell Death Discovery (2022) 8(1):30. doi: 10.1038/s41420-022-00827-7 35046400 PMC8770491

[137] Han D Gao X Wang M Qiao Y Xu Y Yang J. Long noncoding RNA H19 indicates a poor prognosis of colorectal cancer and promotes tumor growth by recruiting and binding to eIF4A3. Oncotarget (2016) 7(16):22159–73. doi: 10.18632/oncotarget.8063 PMC5008352 26989025

[138] Wu DW Lin PL Cheng YW Huang CC Wang L Lee H. DDX3 enhances oncogenic KRAS−induced tumor invasion in colorectal cancer via the beta−catenin/ZEB1 axis. Oncotarget (2016) 7(16):22687–99. doi: 10.18632/oncotarget.8143 PMC5008392 27007150

[139] Wu DW Lin PL Wang L Huang CC Lee H. The YAP1/SIX2 axis is required for DDX3-mediated tumor aggressiveness and cetuximab resistance in KRAS-wild-type colorectal cancer. Theranostics (2017) 7(5):1114–32. doi: 10.7150/thno.18175 PMC5399580 28435452

[140] Fu R Yang P Li Z Liu W Amin S Li Z. Avenanthramide A triggers potent ROS-mediated anti-tumor effects in colorectal cancer by directly targeting DDX3. Cell Death Dis (2019) 10(8):593. doi: 10.1038/s41419-019-1825-5 31391454 PMC6685981

[141] Basu B Karmakar S Basu M Ghosh MK. USP7 imparts partial EMT state in colorectal cancer by stabilizing the RNA helicase DDX3X and augmenting Wnt/beta-catenin signaling. Biochim Biophys Acta Mol Cell Res (2023) 1870(4):119446. doi: 10.1016/j.bbamcr.2023.119446 36791810

[142] Yu P Liang P Pang S Yuan W Zhao Y Huang Q. The function, role and process of DDX58 in heart failure and human cancers. Front Oncol (2022) 12:911309. doi: 10.3389/fonc.2022.911309 35814394 PMC9257035

[143] Song J Zhao W Zhang X Tian W Zhao X Ma L. Mutant RIG-I enhances cancer-related inflammation through activation of circRIG-I signaling. Nat Commun (2022) 13(1):7096. doi: 10.1038/s41467-022-34885-3 36402769 PMC9675819

[144] Cencic R Carrier M Galicia-Vazquez G Bordeleau ME Sukarieh R Bourdeau A. Antitumor activity and mechanism of action of the cyclopenta[b]benzofuran, silvestrol. PloS One (2009) 4(4):e5223. doi: 10.1371/journal.pone.0005223 19401772 PMC2671147

